# Protocol for a mixed-methods exploratory investigation of care following intensive care discharge: the REFLECT study

**DOI:** 10.1136/bmjopen-2018-027838

**Published:** 2019-01-25

**Authors:** Sarah Vollam, Owen Gustafson, Lisa Hinton, Lauren Morgan, Natalie Pattison, Hilary Thomas, J Duncan Young, Peter Watkinson

**Affiliations:** 1 Nuffield Department of Clinical Neurosciences, University of Oxford, Oxford, UK; 2 Adult Intensive Care Unit, Oxford University Hospitals NHS Foundation Trust, Oxford, UK; 3 Nuffield Department of Primary Health Care, University of Oxford, Oxford, UK; 4 Nuffield Department of Surgical Sciences, University of Oxford, Oxford, UK; 5 School of Health and Social Work, University of Hertfordshire, Hatfield, UK; 6 Centre for Research in Public Health and Community Care, University of Hertfordshire, Hatfield, UK; 7 Nuffield Department of Clinical Neurosciences, University of Oxford, Oxford, UK; 8 Nuffield Department of Clinical Neurosciences, University of Oxford, Oxford, UK

**Keywords:** mixed methods, critical care, outcome, protocol

## Abstract

**Introduction:**

A substantial number of patients discharged from intensive care units (ICUs) subsequently die without leaving hospital. It is unclear how many of these deaths are preventable. Ward-based management following discharge from ICU is an area that patients and healthcare staff are concerned about. The primary aim of REFLECT (Recovery Following Intensive Care Treatment) is to develop an intervention plan to reduce in-hospital mortality rates in patients who have been discharged from ICU.

**Methods and analysis:**

REFLECT is a multicentre mixed-methods exploratory study examining ward care delivery to adult patients discharged from ICU. The study will be made up of four substudies. Medical notes of patients who were discharged from ICU and subsequently died will be examined using a retrospective case records review (RCRR) technique. Patients and their relatives will be interviewed about their post-ICU care, including relatives of patients who died in hospital following ICU discharge. Staff involved in the care of patients post-ICU discharge will be interviewed about the care of this patient group. The medical records of patients who survived their post-ICU stay will also be reviewed using the RCRR technique. The analyses of the substudies will be both descriptive and use a modified grounded theory approach to identify emerging themes. The evidence generated in these four substudies will form the basis of the intervention development, which will take place through stakeholder and clinical expert meetings.

**Ethics and dissemination:**

Ethical approval has been obtained through the Wales Research and Ethics Committee 4 (17/WA/0107). We aim to disseminate the findings through international conferences, international peer-reviewed journals and social media.

**Trial registration number:**

ISRCTN14658054.

**Strengths and limitations of this study**

- This exploratory study uses mixed methods to gather rich data from multiple perspectives to inform the development of an intervention.
- This protocol has been designed using Medical Research Council guidance on the development of complex interventions.
- As this is a complex cohort of patients, it is not clear whether problems in care will be distinct enough to be amenable to change through an intervention.

## Introduction

In 2015–2016, over 8000 of the 1 34 000 patients discharged from intensive care units (ICUs) in England and Wales died without leaving hospital.^1^ This mortality rate is higher than hospitalised groups considered to be at high risk^2–4^ and is more than five times the annual number of UK road traffic accident deaths.^5^

Most patients who are discharged from ICU are expected to go home (6 and preliminary analysis provided by Intensive Care National Audit and Research Centre. There are widely varying in-hospital post-ICU mortality rates (2.9% to 22.6%)) for patients of similar illness severity at admission to ICU.^7 8^ Several studies of general ward populations indicate changes in care could lead to improvements in outcome.^9–15^

In 2000, the Department of Health (DH) recognised the need to improve outcomes in this vulnerable patient group, recommending the introduction of critical care outreach ‘to support the continuing recovery of discharged patients on wards …’.^16^ The DH provided substantial financial support to establish these teams. The teams are costly, often constituted of skilled senior critical care practitioners.^17^ However, there is limited evidence in terms of outreach efficacy on reducing mortality in the post-ICU population.^18^

Qualitative studies with patients^19–25^ and staff^26–29^ have identified problems with the transition from ICU to ward care. Many have focused on the psychological impact rather than clinical care, although one study found patients were concerned about the quality and availability of nursing and medical care on the wards.^25^ A secondary analysis of these interviews conducted by the Health Experience Research Group was undertaken as preparatory work for this study (http://www.healthtalk.org). We found patients were able to identify problems in care delivery such as lack of specific clinical skills and awareness of level of physical dependency.

Some studies have investigated which patients are most at risk. Potentially modifiable risk factors identified at ICU discharge include the presence of tracheostomy,^30–32^ elevated C reactive protein^8 33^ or creatinine^33^ and most compellingly, discharge out of hours.^7 34–40^ The evidence identifying risk factors present on the ward after ICU discharge is currently somewhat limited.^41–44^ There have been several single intervention, physical therapy-based strategies which alone have not been found to improve mortality.^45–48^ Recently, the RECOVER study reported no effect from delivering increased physiotherapy and dietetic advice to hospitalised patients following ICU discharge.^49^ The history of interventions tried in this patient group emphasises the need to carefully establish an appropriate intervention package to trial. There is currently insufficient information about the ward management of these patients to know what an effective intervention aimed at reducing post-ICU in-hospital mortality would contain. Recent National Health Service (NHS) guidance^50^ has emphasised the need to incorporate patient experiences to improve their care. In combination with the experience of the carers in the ward environment, evidence from patients provides the most immediate information on identifiable problems with the care they receive. Additionally, case review has previously been shown to yield valuable information with which to improve ward-based care.^9 10 51 52^

The problem is urgent. Over 8000 patients died in 2017 in hospital following discharge from ICU. It is not currently known what proportion of these are expected deaths, but a substantial proportion of these deaths may be avoidable. The operation of ICU outreach teams throughout the country would greatly benefit from the development of an evidence-based care package.

## Methods

### Objectives

Our primary objective is to develop a multifaceted human factors-based intervention to reduce in-hospital mortality rates in patients who have been discharged from ICU. Our secondary objectives are to identify examples of high-quality care and areas for improvement.

### Patient and public involvement

A patient and public involvement (PPI) focus group was conducted during development of this study. The group were consulted on the design of the study with focus on patient/relative interviews approach and the burden of participating. Two members of this group are members of the steering committee. They have been consulted on the ongoing conduct on the study and have provided feedback on participant documentation.

### General design

**Figure 1:**
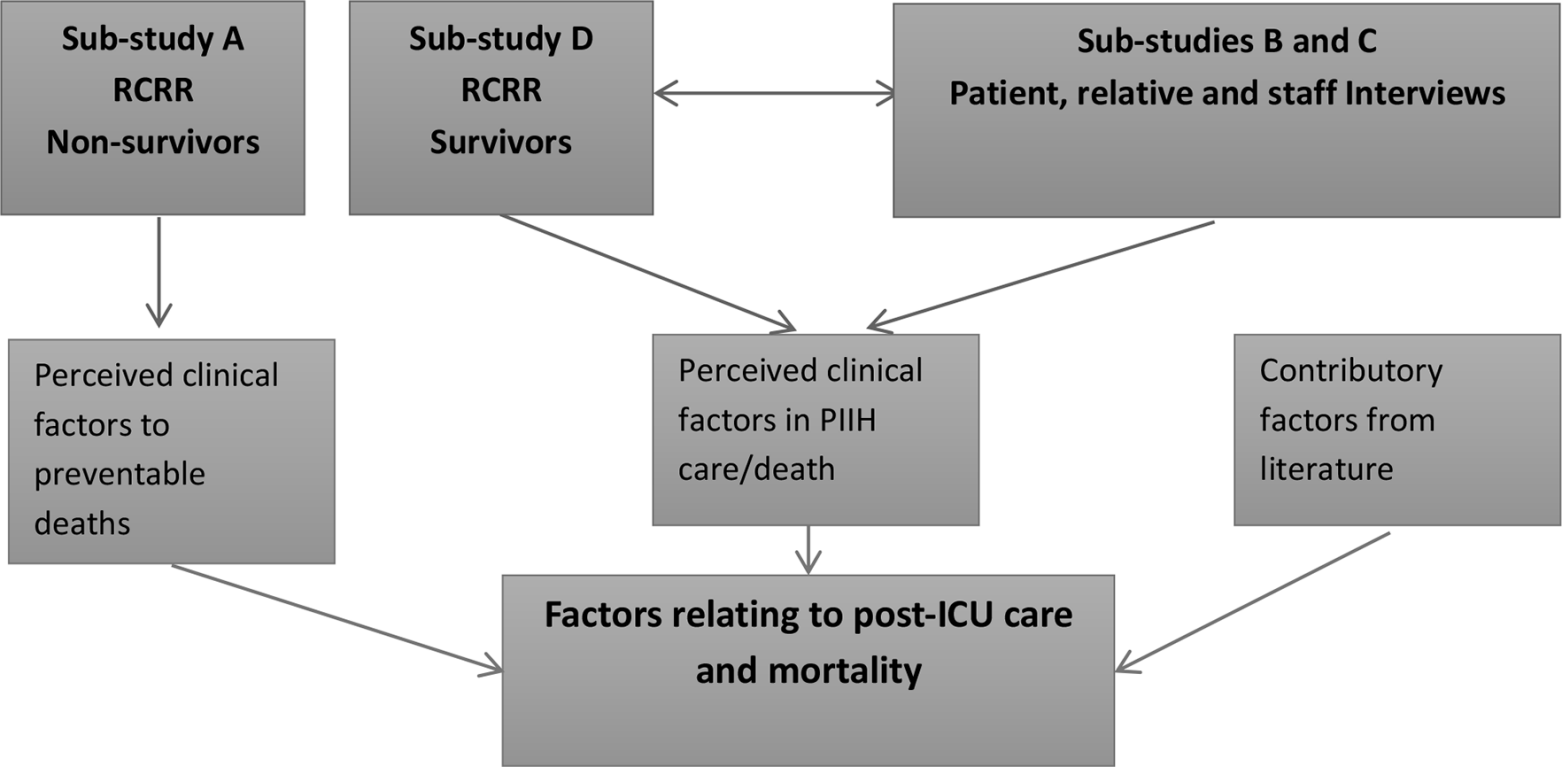
Primary data collection. ICU, intensive care unit; RCRR, retrospective case records review; PIIH, post-intensive care in-hospital.

REFLECT (Recovery Following Intensive Care Treatment) is a multicentre mixed-methods exploratory study examining ward care delivery to patients discharged from ICU. Data collection is split into four substudies: a retrospective case records review (RCRR) of deceased patients, patient and relative interviews/focus groups, staff interviews/focus groups and an RCRR of survivors (figure 1).

#### RCRR deceased patients

Medical notes of patients who were transferred to wards from ICU and subsequently died will be examined using a RCRR technique. This review will use an adaptation of a validated tool for making safety and quality judgements about care delivery.^53–55^ Medical notes are reviewed and ‘structured judgement’ statements are made about the delivery of care. These statements are explicit, value-based comments on care delivery. The output of this is a relatively short but rich account of care delivery, identifying both good and poor care. The output of this stage will be a collation of care delivery, both where it has been excellent and where improvements could be made. This approach has been used extensively in other patient groups,^51 54^ but not previously in this population. It is currently being adopted by the DH as a clinical governance tool within trusts as the National Mortality Care Record Review Programme.^53^ It contains guidance to ensure a consistent and valid approach. We have piloted this review methodology and undertaken preparatory work to ensure the methodology will capture where novel processes could change outcomes for hospitalised patients discharged from ICU. Training will be conducted with the three researchers involved in these reviews, to ensure consistency of findings.

Cases where differences in care delivery could improve outcomes will be further analysed using the ‘change analysis’ method developed by Hogan *et al*.^56^ This is an in-depth qualitative analysis of the narrative account of care delivery for each patient, using a human factors framework. The analysis will allow the identification of areas where novel care processes could change outcomes, and what processes could facilitate this. These findings will guide the design and implementation of the intervention.

#### Patient and relative interviews/focus groups

Patients and their relatives are ideally placed to offer reflection and critique of their care.^57–59^ Our secondary analysis of relative and patient interviews showed patients and relatives could clearly identify areas of their post-ICU ward care which they considered unsatisfactory. However, discussions about post-ICU care were limited as the interviews spanned the entire hospital experience. Further interviews with survivors and their relatives are required to focus on how care on the wards following ICU discharge could be improved. Focus groups will be offered where more than three people are interested in participating on a given day. Telephone interviews will also be offered as an alternative to face-to-face interview.

We will also interview relatives of patients who died in hospital following ICU discharge, to ensure that their experiences are included (involving relatives of patients who died was recommended by our PPI group). This will provide a unique perspective and augment the findings of the RCRR of deceased patients. A focus group or telephone option will not be offered to this group due to the potential for the participant to become distressed, as this would not allow appropriate management of the interview.

#### Staff interviews/focus groups

We will conduct interviews with staff, with focus groups offered where more than three staff members are able to attend together. Interviews/focus groups will be conducted with a variety of staff members to encourage a multidisciplinary analysis of this area of care. Telephone interviews will be offered as an alternative to face to face interviews.

Interviews with patients and staff will be conducted in parallel so that emerging themes can be explored across groups. The interviews will build on themes identified in the preliminary secondary analysis and evidence synthesis discussed above. This work will take an approach informed by the tenets of grounded theory, reflecting the inductive approach to developing an understanding of this area of care.^60 61^ Interviews and focus groups will use a topic guide, based on completed work and input from patient representatives. We anticipate the topic guide will evolve throughout the interviews/focus group phase to ensure any emerging themes are explored,^62^ reflecting the iterative nature of qualitative research.

#### RCRR survivors

We will review the case records of patients who survived their post-ICU ward stay. Ideally, all patients who were interviewed will be included (subject to participant consent). The reviews will follow the same structure proposed for reviewing deceased patient medical notes. This will be modified to assess examples of high-quality care and areas for improvement (using structured judgement and clear rationale). All cases will be further analysed using the ‘change analysis’ method described above. We will triangulate areas identified by patients and relatives with those found in the case records and compare with those identified for non-survivors.

### Study setting

The study is taking place in three separate UK NHS Trusts. There are approximately 2000 patients discharged from the general adult ICUs across the three trusts annually. The RCRR and patient, relative and staff interviews will occur at all three trusts. The specialist cardiothoracic and neurosurgical ICUs will not be included in the study.

### Participant selection

#### RCRR deceased patients

Patients will be identified by a search of the local NHS database. The most recent 300 patients who were discharged from ICU and died during the same hospital admission will be identified and their medical records retrieved. All patients aged 18 years or above discharged from ICU to a ward who died prior to hospital discharge will be included. Any patients with inaccessible medical notes will be excluded.

#### Patient and relative interviews/focus groups

##### Patients discharged from hospital

Patients invited to attend the intensive care follow-up clinic will also be invited to participate in semistructured interviews. Their relatives will also be invited and may participate either as well as or instead of the patient. This invitation will be issued by the clinic organiser (a member of the direct care team). Patients will be eligible if they are willing and able to give informed consent, are 18 years or older and are a patient or relative of a patient who was discharged from ICU to a ward and survived to hospital discharge. Patients will be excluded if they lack the capacity to consent or have poor spoken English as it will not be possible to conduct the interviews through an interpreter. Participants will be sought with varying experiences, to facilitate maximum variation in the sample.^63^

##### Patients who did not survive to hospital discharge

Our planned involvement of relatives of patients who died follows advice from two experts in the field, Dr Colin Parkes (emeritus Senior Lecturer in Psychiatry, Royal London Hospital) and Professor Maggie Stroebe-Harrold (University of Utrecht), published guidelines,^64^ bereavement research^65^ and advice from the study PPI group. A pack will be sent by the ICU follow-up team to relatives of patients who were discharged from ICU and subsequently died on a ward. This will include a covering letter, brief leaflet and participant information sheet. Letters will be sent out 6 months following the relative’s death, as suggested by bereavement research.^64 65^ The letter will invite the relative to consider the study and contact the study team if they are interested. It will clearly state that they are very welcome to completely discard the letter and no further contact will be made. It will also be made clear that if they do participate, they can withdraw at any time, including during the interview.

If we are unable to recruit participants through this approach, we may contact local support groups, such as ICUSteps (www.icusteps.org) to explore recruitment through them. The study has been endorsed by the national ICUSteps group. In this instance, packs (including covering letter, leaflet and PIS) would be given out by the group facilitator if, and when, they felt this was appropriate. This direct approach is used successfully by the Health Experience Research Group in many of their studies, including those recruiting bereaved relatives.^25 66^ Participants will be included if they are willing and able to give informed consent, are 18 years or older and are a relative of a patient who was discharged from ICU and did not survive to hospital discharge. As with survivor interviews, participants will be excluded if they lack the capacity to consent or have poor spoken English.

#### Staff interviews/focus groups

Staff involved in the care of patients discharged from ICU to the wards (including nurses, doctors, physiotherapists, dieticians and other allied health professionals) will be recruited to participate in interviews/focus groups. As above, purposive sampling will be used to ensure a diverse range of exposure, experience and background training. Invitation letters and attached participant information sheets will be distributed to all staff by ward clerks, or a similar member of staff to wards with a high throughput of post-ICU patients. In addition, posters will be placed on wards, advertisements placed on trust-wide intranet and prior contact with senior managers will be sought for endorsement. We also anticipate an element of snowballing from other participants. Participants will be included if they are willing and able to give informed consents, are aged 18 years or older and are a member of NHS staff involved in the care of patients discharged from ICU to the wards. There are no exclusion criteria.

#### RCRR survivors

Patients who are approached to participate in the interview study will also be asked to participate in the RCRR. Ideally, all those who are interviewed will consent to notes review, but it is anticipated that some may not. Patients may consent to the RCRR without participating in the interview study. Information about the study will be sent out with the ICU follow-up clinic appointment, around 2 weeks in advance. Participants will be included if they are willing and able to give informed consent, are aged 18 years or older and have been discharged from ICU to the ward and subsequently discharged from hospital.

### Consent

Consent will not be obtained for the RCRR for deceased patients. Support to access notes for this group will be sought from the Confidentiality Advisory Group, who advise the Health Research Authority on applications to process patient information without consent. For patients/relatives undertaking interviews, consent will be sought by trained researchers at the time of interview if face-to-face. Postal consent will be offered as an alternative if the participant requests a telephone interview or for notes review only. If the patient opts for notes review only, they may sign and return the consent form without speaking with the research team. The patient will be able to discuss the study with a member of the study team prior to signing the consent form if they wish. Documents relating to informed consent are available within the trial registry.

### Sample size

#### RCRR deceased patients

Based on previous audit, up to 300 patient records will be reviewed, yielding approximately 30 records for in-depth analysis. These records will be sourced from all three trusts.

#### Patient and relative interviews

We estimate approximately 20 interviews will be required to supplement data from our secondary analysis of patient and relative interviews. We anticipate these participants will be recruited from all three trusts. Data collection will continue with concurrent thematic analysis, until theoretical saturation has been reached (ie, no new themes are emerging). Anticipated numbers are given for each group, but may vary to achieve saturation.^60 61^

#### Staff interviews

we anticipate conducting interviews/focus groups with approximately 30 staff members, across all three trusts.

#### RCRR survivors

Up to 30 patient records (to match the number for in-depth analysis above). We anticipate these will be recruited from across the three trusts.

### Data storage

All electronic data will be password-protected and stored on a secure server within a university research facility. All paper documentation (such as consent forms and case report forms) will be stored in a locked university research facility behind two swipe access doors.

### Data analysis

#### RCRR deceased and survivors

Statistical analysis will be mostly descriptive. This will include proportions of patients experiencing one or more ‘problem with care’. For deceased patients, we will report the proportion of cases deemed to have more than a 50% chance of death being avoidable. Avoidability will be judged based on the case record review and decisions discussed and verified between the three researchers conducting the RCRR. For survivors we will report proportion of cases who experienced examples of high-quality care and areas where improvements could be made. Cases where improvements could be made (perhaps using examples of high-quality care) will be further analysed using the ‘change analysis’ method developed by Hogan *et al*.^56^ This additional analysis will add an in-depth qualitative analysis of the links between identified ‘care areas’ and associated human factors. This is particularly useful in cases with multiple complex problems, anticipated to be the case in this population.

We will triangulate ‘care areas’ identified by patients and relatives with those found in the case records. We will compare the ‘care areas’ identified with those identified for non-survivors. Records will be reviewed after interview, to avoid any potential conflict of interest for the researcher.

A report will be produced summarising the potential areas and approaches for interventions and the human factors which contributed to the identified ‘care areas’.

#### Interviews and focus groups

Audio recordings will be transcribed verbatim and entered into qualitative analysis software (NVivo). Interviews and focus groups will be transcribed verbatim into a specialist software package for coding qualitative data (QSR NVivo). A modified grounded theory approach will be used to identify emerging themes. This will ensure identification of ‘care areas’ important to patients and health professionals, as well as those that researchers anticipate.^60 61 67^ This approach has previously been used to identify areas of care which patients believed could be improved.^25 68 69^

Preliminary coding will take place soon after the interviews are conducted. This will allow any emerging themes to be explored in subsequent interviews. Preliminary coding will be refined using the method of constant comparison (until no new themes emerge) to produce a report for each theme.^60^ Each report will reflect the most important themes that participants talk about in their interviews and represent the full range of experiences included in the interviews. These reports will reviewed and themes will be verified within the research team, comprising four qualitative researchers (SV, HT, NP and LH).^69^ Any differences in interpretation or emphasis will be discussed and resolved. For the final output, these themes will be further categorised by areas of care which could be improved, and suggestions for improvement.

### Modelling the intervention

#### Stakeholder meeting

**Figure 2:**
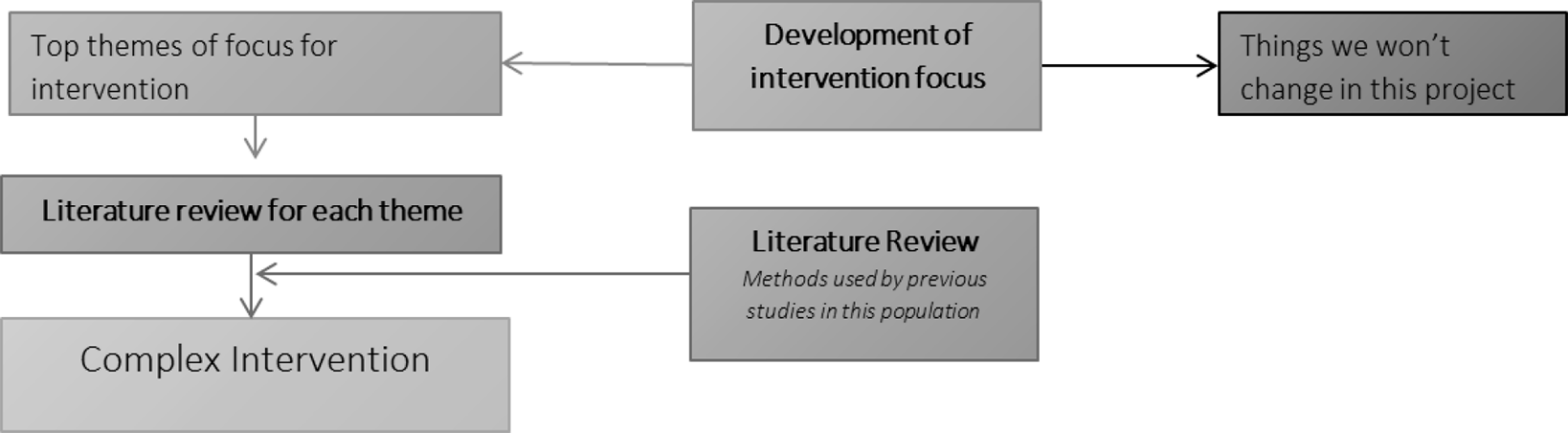
Modelling the intervention.

The evidence generated through the methodology above will form the basis of the intervention development (figure 2). Guided by a Human Factors researcher, a stakeholder group will prioritise areas for intervention from those identified in the interviews, focus groups, case record reviews and our earlier research. The meeting will take the form of a prioritisation exercise, including a facilitated card sort to rank the potential areas for improvement. They will select the most promising areas that can be pragmatically combined in a multifaceted intervention. For an area to be prioritised, the mechanism by which intervention in that area could be expected to reduce mortality will need to be defined.

#### Literature searches

We will then undertake literature searches to check if our prioritised areas have been previously investigated in other hospitalised patient populations. To capture relevant successful methods for change implementation, we will review previous implementation methods for interventions in the post-ICU hospitalised patient group and methods used in studies of our prioritised areas in other hospitalised patient populations. This will result in a refined list of areas for inclusion and identification of previous methods used to successfully implement change in these areas.

#### Paper modelling exercise

Components of the multifaceted intervention will be examined in an initial paper modelling exercise.^70^ This exercise will allow exploration of: the interdependencies of the components, different implementation strategies and challenges that may be encountered.

#### Clinical experts meeting

The prioritised areas and the results of the paper modelling exercise will be taken to meeting of stakeholders and clinical experts. At this meeting, the proposed intervention will be finalised with input from those likely to deliver the intervention and those who have previously experienced care.

## Ethics and dissemination

### Ethics

The study has received ethical approval from the Wales Research Ethics Committee. The University of Oxford will act as sponsor. The study will be overseen by a steering committee and includes PPI involvement throughout.

This paper reports protocol version 1 (April 2017) and has been written with reference to the SPIRIT (Standard Protocol Items: Recommendations for Interventional Trials) checklist.^71^

#### RCRR deceased patients

As informed consent cannot be obtained for deceased patients in this substudy, an application has been approved by the Confidentiality Advisory Group for suspension of the duty of confidentiality under Section 251 of the NHS Act 2006 specifically in relation to this section of the project. The research brings the possibility of identification of areas where practice may not have been optimal, which will be referred through the organisations standard clinical governance processes. The response will follow the guidance given by the Royal College of Physicians Clinical governance guide to mortality case record reviews.^53^

#### Patient and relative interviews

Where possible, for patients, these interviews/focus groups will take place on the same day as their ICU follow-up clinic appointment. This will ensure support will be available should the interview raise issues that may cause distress. For patients and relatives requiring further support, appropriate referrals will be made within the existing hospital system and details of organisations outside the hospital offered.

Relatives of deceased patients will be identified and sensitively approached as discussed above. Training on talking with bereaved relatives will be provided for researchers. We will also use the ‘buddy’ system used by the Health Experiences Research Group, whereby another researcher will be available to debrief after each interview if necessary.

#### Staff interviews/focus groups

Given the sensitive nature of this subject, it is possible that discussions may cause distress to staff members. NHS Trust Occupational Health will be made aware that we are conducting this study and any staff member who causes concern to the researchers will be signposted to occupational health in the first instance.

Any answers which cause concern in terms of professional conduct will be discussed with clinicians within their management structure in the first instance, with a view to raising this with the line manager of the participant. Any disclosures raising serious concerns about a specific patient will be dealt with as described above.

#### RCRR survivors

It is anticipated that most patients participating in the RCRR will also be interviewed. In order to ensure there is no bias or conflict of interest which might influence the conversation, these reviews will be completed after the interviews. Any identified significant care areas will be escalated as outlined for the RCRR for deceased patients.

### Dissemination

Results from this study will be disseminated at regional and international conferences and in peer-reviewed journals. Authorship of any papers related to this study will follow the ICMJE recommendations (http://www.icmje.org/recommendations/).

## Supplementary Material

Reviewer comments

Author's manuscript
