# Supplementary material for: Protocol for a mixed-methods exploratory investigation of care following intensive care discharge: the REFLECT study
Source: BMJ Open. 2019 Jan 25;9(1):e027838. doi: 10.1136/bmjopen-2018-027838 (PMC6347880; doi:10.1136/bmjopen-2018-027838)
Supplement: Author's manuscript [file bmjopen-2018-027838.draft_revisions.pdf]

BMJ Open is committed to open peer review. As part of this commitment we make the peer review history of every article we publish publicly available.

When an article is published we post the peer reviewers' comments and the authors' responses online. We also post the versions of the paper that were used during peer review. These are the versions that the peer review comments apply to.

The versions of the paper that follow are the versions that were submitted during the peer review process. They are not the versions of record or the final published versions. They should not be cited or distributed as the published version of this manuscript.

BMJ Open is an open access journal and the full, final, typeset and author-corrected version of record of the manuscript is available on our site with no access controls, subscription charges or pay-per-view fees (http://bmjopen.bmj.com).

If you have any questions on BMJ Open's open peer review process please email info.bmjopen@bmj.com

BMJ Open

Protocol for a mixed methods exploratory investigation of care following intensive care discharge: the REFLECT study.

Journal:	*BMJ Open*
Manuscript ID	bmjopen-2018-027838
Article Type:	Protocol
Date Submitted by the Author:	09-Nov-2018
Complete List of Authors:	Vollam, Sarah; University of Oxford, Nuffield Department of Neurosciences<br>Gustafson, Owen; Oxford University Hospitals NHS Foundation Trust, Adult Intensive Care Unit<br>Hinton, Lisa; University of Oxford, Health Experiences Research Group, Nuffield Department of Primary Care Health Sciencs<br>Morgan, Lauren; University of Oxford, Nuffield Department of Surgical Sciences<br>Pattison, Natalie; University of Hertfordshire, School of Health and Social Work<br>Thomas, Hilary; University of Hertfordshire, CRIPACC<br>Young, Duncan; Oxford University, Nuffield Department of Clinical Neurosciences<br>Watkinson, Peter; University of Oxford, Nuffield Department of Clinical Neurosciences
Keywords:	Mixed methods, Critical Care, Outcome, Protocol

SCHOLARONE™
Manuscripts

Protocol for a mixed methods exploratory investigation of care following intensive care discharge: the REFLECT study.

Sarah Vollam (Corresponding author)
Nuffield Department of Clinical Neurosciences
University of Oxford
Level 6, West Wing, John Radcliffe Hospital
Oxford
OX3 9DU
UK
Sarah.vollam@ndcn.ox.ac.uk
01865 231440

Owen Gustafson
Adult Intensive Care Unit
Oxford University Hospitals NHS Foundation Trust
Oxford
UK

Dr Lisa Hinton
Nuffield Department of Primary Health Care
University of Oxford
Oxford
UK

Dr Lauren Morgan
Nuffield Department of Surgical Sciences
University of Oxford
Oxford
UK

Prof Natalie Pattison
School of Health and Social Work
University of Hertfordshire
Hatfield
UK

Prof Hilary Thomas
Centre for Research in Public Health and Community Care
University of Hertfordshire
Hatfield
UK

J. Duncan Young
Nuffield Department of Clinical Neurosciences
University of Oxford
Oxford
UK

Assoc Prof Peter Watkinson
Nuffield Department of Clinical Neurosciences
University of Oxford
Oxford
UK

Word count: 3,954

For peer review only

For peer review only - http://bmjopen.bmj.com/site/about/guidelines.xhtml

ABSTRACT

Introduction

A substantial number of patients discharged from Intensive Care Units (ICUs) subsequently die without leaving hospital. It is unclear how many of these deaths are preventable. Ward-based management following discharge from ICU is an area that patients and healthcare staff are concerned about. The primary aim of REFLECT (Recovery Following Intensive Care Treatment) is to develop an intervention plan to reduce in-hospital mortality rates in patients who have been discharged from ICU.

Methods and analysis

REFLECT is a multicentre mixed methods exploratory study examining ward care delivery to adult patients discharged from ICU. The study will be made up of four sub-studies. Medical notes of patients who were discharged from intensive care and subsequently died will be examined using a Retrospective Case Records Review (RCRR) technique. Patients and their relatives will be interviewed about their post-ICU care, including relatives of patients who died in hospital following ICU discharge. Staff involved in the care of patients post-ICU discharge will be interviewed about the care of this patient group. The medical records of patients who survived their post-ICU stay will also be reviewed using the RCRR technique. The analyses of the sub-studies will be both descriptive and use a modified grounded theory approach to identify emerging themes. The evidence generated in these four sub-studies will form the basis of the intervention development, which will take place through stakeholder and clinical expert meetings.

Ethics and dissemination

Ethical approval has been obtained through the Wales Research and Ethics Committee 4 (17/WA/0107). We aim to disseminate the findings through international conferences, international peer-reviewed journals and social media.

Trial registration number

ISRCTN14658054

For peer review only

ARTICLE SUMMARY

Strengths and limitations of this study

- This exploratory study uses mixed methods to gather rich data from multiple perspectives to inform the development of an intervention.
- This protocol has been designed using MRC guidance on the development of complex interventions.
- As this is a complex cohort of patients, it is not clear whether problems in care will be distinct enough to be amenable to change through an intervention.

For peer review only - http://bmjopen.bmj.com/site/about/guidelines.xhtml

INTRODUCTION

In 2015-16, over 8000 of the 134,000 patients discharged from Intensive Care Units (ICUs) in England and Wales died without leaving hospital.[1] This mortality rate is higher than hospitalised groups considered to be at high risk [2–4] and is more than five times the annual number of UK road traffic accident deaths.[5]

Most patients discharged from ICU are expected to go home ([6] and preliminary analysis provided by Intensive Care National Audit and Research Centre. There are widely varying in-hospital post-ICU mortality rates (2.9% to 22.6%) for patients of similar illness severity at admission to ICU.[7,8] Several studies of general ward populations indicate changes in care could lead to improvements in outcome.[9–15]

In 2000, the Department of Health (DH) recognised the need to improve outcomes in this vulnerable patient group, recommending the introduction of critical care outreach "to support the continuing recovery of discharged patients on wards…".[16] The DH provided substantial financial support to establish these teams. The teams are costly, often constituted of skilled senior critical care practitioners.[17] However, there is limited evidence in terms of outreach efficacy on reducing mortality in the post-ICU population.[18]

Qualitative studies with patients [19–25] and staff [26–29] have identified problems with the transition from ICU to ward care. Many have focused on the psychological impact rather than clinical care, although one study found patients were concerned about the quality and availability of nursing and medical care on the wards.[25] A secondary analysis these interviews conducted by the Health Experience Research Group was undertaken as preparatory work for this study (http://www.healthtalk.org). We found patients were able to identify problems in care delivery such as lack of specific clinical skills and awareness of level of physical dependency.

Some studies have investigated which patients are most at risk. Potentially modifiable risk factors identified at ICU discharge include the presence of tracheostomy, [30–32] elevated C-reactive protein [8,33-34] or creatinine [34] and most compellingly, discharge out of hours [7,35–42]. The evidence identifying risk factors present on the ward after ICU discharge is currently somewhat limited.[43–46] There have been several single intervention, physical therapy-based strategies which alone have not been found to improve mortality.[47–50] Recently the RECOVER study reported no effect from delivering increased physiotherapy and dietetic advice to hospitalised patients following ICU discharge.[51] The history of interventions tried in this patient group emphasises the need to carefully establish an appropriate intervention package to trial. There is currently insufficient information about the ward management of these patients to know what an effective intervention aimed at reducing post-ICU in-hospital mortality would contain. Recent NHS guidance [52] has emphasised the need to incorporate patient experiences to improve their care. In combination with the experience of the carers in the ward environment, evidence from patients provides the most immediate information on identifiable problems with the care they receive. Additionally, case review has previously been shown to yield valuable information with which to improve ward-based care. [9,10,53-54]

The problem is urgent. Over 8,000 patients died in 2017 in hospital following discharge from intensive care. It is not currently known what proportion of these are expected deaths, but a substantial proportion of these deaths may be avoidable. The operation of ICU outreach teams throughout the country would greatly benefit from the development of an evidence-based care package.

METHODS

For peer review only - http://bmjopen.bmj.com/site/about/guidelines.xhtml

Objectives

Our primary objective is to develop a multifaceted human factors-based intervention to reduce in-hospital mortality rates in patients who have been discharged from intensive care. Our secondary objectives are to identify examples of high-quality care and areas for improvement.

Patient and Public Involvement

A patient and public (PPI) focus group was conducted during development of this study. The group were consulted on the design of the study with focus on patient/relative interviews approach and the burden of participating. Two members of this group are members of the steering committee. They have been consulted on the ongoing conduct on the study and have provided feedback on participant documentation.

General design

REFLECT is a multicentre mixed methods exploratory study examining ward care delivery to patients discharged from intensive care. Data collection is split into four sub-studies: a retrospective case records review (RCRR) of deceased patients, patient and relative interviews/focus groups, staff interviews/focus groups, and a RCRR of survivors (Figure 1).

RCRR deceased patients

Medical notes of patients who were transferred to wards from ICU and subsequently died will be examined using a Retrospective Case Record Review (RCRR) technique. This review will use an adaptation of a validated tool for making safety and quality judgements about care delivery.[55–57] Medical notes are reviewed and 'structured judgement' statements are made about the delivery of care. These statements are explicit, value-based comments on care delivery. The output of this is a relatively short but rich account of care delivery, identifying both good and poor care. The output of this stage will be a collation of care delivery, both where it has been excellent and where improvements could be made. This approach has been used extensively in other patient groups,[53,56] but not previously in this population. It is currently being adopted by the DH as a clinical governance tool within trusts as the National Mortality Care Record Review Programme.[55] It contains guidance to ensure a consistent and valid approach. We have piloted this review methodology and undertaken preparatory work to ensure the methodology will capture where novel processes could change outcomes for hospitalised patients discharged from Intensive Care. Training will be conducted with the three researchers involved in these reviews, to ensure consistency of findings.

Cases where differences in care delivery could improve outcomes will be further analysed using the 'change analysis' method developed by Hogan et al.[58] This is an in-depth qualitative analysis of the narrative account of care delivery for each patient, using a human factors framework. The analysis will allow identification of areas where novel care processes could change outcomes, and what processes could facilitate this. These findings will guide the design and implementation of the intervention.

Patient and relative interviews/focus groups

Patients and their relatives are ideally placed to offer reflection and critique of their care.[59–61] Our secondary analysis of relative and patient interviews showed patients and relatives could clearly identify areas of their post-ICU ward care which they considered unsatisfactory. However, discussions about post-ICU care were limited as the interviews spanned the entire hospital

For peer review only - http://bmjopen.bmj.com/site/about/guidelines.xhtml

experience. Further interviews with survivors and their relatives are required to focus on how care on the wards following ICU discharge could be improved. Focus groups will be offered where more than three people are interested in participating on a given day. Telephone interviews will also be offered as an alternative to face-to-face interview.

We will also interview relatives of patients who died in hospital following intensive care discharge, to ensure that their experiences are included (involving relatives of patients who died was recommended by our patient and public involvement (PPI) group). This will provide a unique perspective and augment the findings of the RCRR of deceased patients. A focus group or telephone option will not be offered to this group due to the potential for the participant to become distressed, as this would not allow appropriate management of the interview.

Staff interviews/focus groups

We will conduct interviews with staff, with focus groups offered where more than three staff members are able to attend together. Interviews/focus groups will be conducted with a variety of staff members to encourage a multi-disciplinary analysis of this area of care. Telephone interviews will be offered as an alternative to face to face interviews.

Interviews with patients and staff will be conducted in parallel so that emerging themes can be explored across groups. The interviews will build on themes identified in the preliminary secondary analysis and evidence synthesis discussed above. This work will take an approach informed by the tenets of grounded theory, reflecting the inductive approach to developing an understanding of this area of care.[62-63] Interviews and focus groups will use a topic guide, based on completed work and input from patient representatives. We anticipate the topic guide will evolve throughout the interviews/focus group phase to ensure any emerging themes are explored,[64] reflecting the iterative nature of qualitative research.

RCRR survivors

We will review the case records of patients who survived their post-ICU ward stay. Ideally, all patients who were interviewed will be included (subject to participant consent). The reviews will follow the same structure proposed for reviewing deceased patient medical notes. This will be modified to assess examples of high quality care and areas for improvement (using structured judgement and clear rationale). All cases will be further analysed using the ‘change analysis’ method described above. We will triangulate areas identified by patients and relatives with those found in the case records and compare with those identified for non-survivors.

Study setting

The study is taking place in three separate United Kingdom NHS Trusts. There are approximately 2000 patients discharged from the general adult ICUs across the three trusts annually. The RCRR and patient, relative and staff interviews will occur at all three trusts. The specialist cardiothoracic and neurosurgical ICUs will not be included in the study.

Participant selection

RCRR deceased patients

Patients will be identified by a search of the local NHS database. The most recent 300 patients who were discharged from ICU and died during the same hospital admission will be identified and their medical records retrieved. All patients aged 18 years or above discharged from ICU to a ward who

For peer review only - http://bmjopen.bmj.com/site/about/guidelines.xhtml

died prior to hospital discharge will be included. Any patients with inaccessible medical notes will be excluded.

Patient and relative interviews/focus groups

Patients discharged from hospital: patients invited to attend the intensive care follow-up clinic will also be invited to participate in semi-structured interviews. Their relatives will also be invited and may participate either as well as or instead of the patient. This invitation will be issued by the clinic organiser (a member of the direct care team). Patients will be eligible if they are willing and able to give informed consent, are 18 years or older and are a patient or relative of a patient who was discharged from ICU to a ward and survived to hospital discharge. Patients will be excluded if they lack the capacity to consent or have poor spoken English as it will not be possible to conduct the interviews through an interpreter. Participants will be sought with varying experiences, to facilitate maximum variation in the sample.[65]

Patients who did not survive to hospital discharge: our planned involvement of relatives of patients who died follows advice from two experts in the field, Dr Colin Parkes (emeritus Senior Lecturer in Psychiatry, Royal London Hospital) and Prof Maggie Stroebe-Harrold (University of Utrecht), published guidelines,[66] bereavement research,[67] and advice from the study PPI group. A pack will be sent by the ICU follow-up team to relatives of patients who were discharged from ICU and subsequently died on a ward. This will include a covering letter, brief leaflet and Participant Information Sheet. Letters will be sent out 6 months following the relative's death, as suggested by bereavement research.[66-67] The letter will invite the relative to consider the study and contact the study team if they are interested. It will clearly state that they are very welcome to completely discard the letter and no further contact will be made. It will also be made clear that if they do participate, they can withdraw at any time, including during the interview.

If we are unable to recruit participants through this approach we may contact local support groups, such as ICUSteps (www.icusteps.org) to explore recruitment through them. The study has been endorsed by the national ICUSteps group. In this instance, packs (including covering letter, leaflet and PIS) would be given out by the group facilitator if, and when, they felt this was appropriate. This direct approach is used successfully by the Health Experience Research Group in many of their studies, including those recruiting bereaved relatives.[25,68] Participants will be included if they are willing and able to give informed consent, are 18 years or older and are a relative of a patient who was discharged from ICU and did not survive to hospital discharge. As with survivor interviews, participants will be excluded if they lack the capacity to consent or have poor spoken English.

Staff interviews/focus groups

Staff involved in the care of patients discharged from ICU to the wards (including nurses, doctors, physiotherapists, dieticians and other allied health professionals) will be recruited to participate in interviews/focus groups. As above, purposive sampling will be utilised to ensure a diverse range of exposure, experience and background training. Invitation letters and attached participant information sheets will be distributed to all staff by ward clerks, or a similar member of staff to wards with a high throughput of post-ICU patients. In addition: posters will be placed on wards, advertisements placed on trust-wide intranet and prior contact with senior managers will be sought for endorsement. We also anticipate an element of snowballing from other participants. Participants will be included if they are willing and able to give informed consents, are aged 18 years or older and are a member of NHS staff involved in the care of patients discharged from ICU to the wards. There are no exclusion criteria.

For peer review only - http://bmjopen.bmj.com/site/about/guidelines.xhtml

RCRR survivors

Patients who are approached to participate in the interview study will also be asked to participate in the RCRR. Ideally, all those who are interviewed will consent to notes review, but it is anticipated that some may not. Patients may consent to the RCRR without participating in the interview study. Information about the study will be sent out with the ICU follow-up clinic appointment, around two weeks in advance. Participants will be included if they are willing and able to give informed consent, are aged 18 years or older and have been discharged from ICU to the ward and subsequently discharged from hospital.

Consent

Consent will not be obtained for the RCRR for deceased patients. Support to access notes for this group will be sought from the Confidentiality Advisory Group, who advise the Health Research Authority on applications to process patient information without consent. For patients/relatives undertaking interviews, consent will be sought by trained researchers at the time of interview if face-to-face. Postal consent will be offered as an alternative if the participant requests a telephone interview or for notes review only. If the patient opts for notes review only, they may sign and return the consent form without speaking with the research team. The patient will be able to discuss the study with a member of the study team prior to signing the consent form if they wish. Documents relating to informed consent are available within the trial registry.

Sample size

RCRR deceased patients: based on previous audit, up to 300 patient records will be reviewed, yielding approximately 30 records for in-depth analysis. These records will be sourced from all three trusts.

Patient and relative interviews: we estimate approximately 20 interviews will be required to supplement data from our secondary analysis of patient and relative interviews. We anticipate these participants will be recruited from all three trusts. Data collection will continue with concurrent thematic analysis, until theoretical saturation has been reached (i.e. no new themes are emerging). Anticipated numbers are given for each group, but may vary to achieve saturation.[62-63]

Staff interviews: we anticipate conducting interviews/focus groups with approximately 30 staff members, across all three trusts.

RCRR survivors: up to 30 patient records (to match the number for in-depth analysis above). We anticipate these will be recruited from across the three trusts.

Data storage

All electronic data will be password-protected and stored on a secure server within a university research facility. All paper documentation (such as consent forms and case report forms) will be stored in a locked university research facility behind two swipe access doors.

Data analysis

RCRR deceased and survivors

Statistical analysis will be mostly descriptive. This will include proportions of patients experiencing one or more ‘problem with care’. For deceased patients, we will report the proportion of cases deemed to have more than a 50% chance of death being avoidable. Avoidability will be judged based

For peer review only

on the case record review and decisions discussed and verified between the three researchers conducting the RCRR. For survivors we will report proportion of cases who experienced examples of high-quality care and areas where improvements could be made. Cases where improvements could be made (perhaps using examples of high quality care) will be further analysed using the 'change analysis' method developed by Hogan et al.[58] This additional analysis will add an in-depth qualitative analysis of the links between identified 'care areas' and associated human factors. This is particularly useful in cases with multiple complex problems, anticipated to be the case in this population.

We will triangulate "care areas" identified by patients and relatives with those found in the case records. We will compare the 'care areas' identified with those identified for non-survivors. Records will be reviewed after interview, to avoid any potential conflict of interest for the researcher.

A report will be produced summarising the potential areas and approaches for interventions and the human factors which contributed to the identified "care areas".

Interviews and focus groups

Audio recordings will be transcribed verbatim and entered into qualitative analysis software (NVivo). Interviews and focus groups will be transcribed verbatim into a specialist software package for coding qualitative data (QSR NVivo). A modified grounded theory approach will be used to identify emerging themes. This will ensure identification of "care areas"   important to patients and health professionals, as well as those that researchers anticipate.[62-63,69] This approach has previously been used to identify areas of care which patients believed could be improved.[25,70-71]

Preliminary coding will take place soon after the interviews are conducted. This will allow any emerging themes to be explored in subsequent interviews. Preliminary coding will be refined using the method of constant comparison (until no new themes emerge) to produce a report for each theme.[62] Each report will reflect the most important themes that participants talk about in their interviews and represent the full range of experiences included in the interviews. These reports will reviewed and themes will be verified within the research team, comprising of four qualitative researchers (SV, HT, NP and LH).[71] Any differences in interpretation or emphasis will be discussed and resolved. For the final output, these themes will be further categorised by areas of care which could be improved, and suggestions for improvement.

Modelling the Intervention

Stakeholder meeting

The evidence generated through the methodology above, will form the basis of the intervention development (Figure 2). Guided by a Human Factors researcher, a stakeholder group will prioritise areas for intervention from those identified in the interviews, focus groups, case record reviews and our earlier research. The meeting will take the form of a prioritisation exercise, including a facilitated card sort to rank the potential areas for improvement. They will select the most promising areas that can be pragmatically combined in a multi-faceted intervention. For an area to be prioritised, the mechanism by which intervention in that area could be expected to reduce mortality will need to be defined.

Literature searches

We will then undertake literature searches to check if our prioritised areas have been previously investigated in other hospitalised patient populations. To capture relevant successful methods for

For peer review only - http://bmjopen.bmj.com/site/about/guidelines.xhtml

change implementation we will review previous implementation methods for interventions in the post-ICU hospitalised patient group and methods used in studies of our prioritised areas in other hospitalised patient populations. This will result in a refined list of areas for inclusion and identification of previous methods used to successfully implement change in these areas.

Paper modelling exercise

Components of the multi-faceted intervention will be examined in an initial paper modelling exercise.[72] This exercise will allow exploration of: the interdependencies of the components, different implementation strategies and challenges that may be encountered.

Clinical experts meeting

The prioritised areas and the results of the paper modelling exercise will be taken to meeting of stakeholders and clinical experts. At this meeting the proposed intervention will be finalised with input from those likely to deliver the intervention and those who have previously experienced care.

ETHICS AND DISSEMINATION

Ethics

The study has received ethical approval from the Wales Research Ethics Committee (Ref: 17/WA/0107). The University of Oxford will act as sponsor. The study will be overseen by a steering committee and includes PPI involvement throughout.

This trial is registered: ISRCTN14658054. This paper reports protocol version 1 (April 2017), and has been written with reference to the SPIRIT checklist.[72]

RCRR deceased patients

As informed consent cannot be obtained for deceased patients in this sub-study, an application has been approved by the Confidentiality Advisory Group for suspension of the duty of confidentiality under section 251 of the NHS Act 2006 specifically in relation to this section of the project. The research brings the possibility of identification of areas where practice may not have been optimal, which will be referred through the organisations standard clinical governance processes. The response will follow the guidance given by the Royal College of Physicians Clinical governance guide to mortality case record reviews.[55]

Patient and relative interviews

Where possible, for patients, these interviews/focus groups will take place on the same day as their ICU follow up clinic appointment, where support will be available should the interview raise issues that may cause distress. For patients and relatives requiring further support appropriate referrals will be made within the existing hospital system and details of organisations outside the hospital offered.

Relatives of deceased patients will be identified and sensitively approached as discussed above. Training on talking with bereaved relatives will be provided for researchers. We will also use the ‘buddy’ system utilised by the Health Experiences Research Group, whereby another researcher will be available to debrief after each interview if necessary.

Staff interviews/focus groups

For peer review only - http://bmjopen.bmj.com/site/about/guidelines.xhtml

Given the sensitive nature of this subject, it is possible that discussions may cause distress to staff members. NHS Trust Occupational Health will be made aware that we are conducting this study and any staff member who causes concern to the researchers will be signposted to occupational health in the first instance.

Any answers which cause concern in terms of professional conduct will be discussed with clinicians within their management structure in the first instance, with a view to raising this with the line manager of the participant. Any disclosures raising serious concerns about a specific patient will be dealt with as described above.

RCRR survivors

It is anticipated that most patients participating in the RCRR will also be interviewed. In order to ensure there is no bias or conflict of interest which might influence the conversation, these reviews will be completed after the interviews. Any identified significant care areas will be escalated as outlined for the RCRR for deceased patients.

Dissemination

Results from this study will be disseminated at regional and international conferences and in peer-reviewed journals. Authorship of any papers related to this study will follow the ICMJE recommendations (http://www.icmje.org/recommendations/).

Data sharing

Consent was given by participants for anonymised data to be made available to other researchers undertaking relevant research. Applications to use anonymised data will be considered by the steering committee.

CONTRIBUTORS

SV, PW and DY conceived the project. SV, PW, DY, HT, LM and LH developed the protocol. PW, NP and HT are providing PhD supervision for SV and supporting data analysis. All authors contributed to and revised the final manuscript.

FUNDING STATEMENT

This paper presents independent research funded by the National Institute for Health Research (NIHR) under its Research for Patient Benefit (RfPB) Programme (Grant Reference Number PB-PG-0215-36149). The views expressed are those of the author(s) and not necessarily those of the NHS, the NIHR or the Department of Health.

COMPETING INTERESTS

None declared.

For peer review only - http://bmjopen.bmj.com/site/about/guidelines.xhtml

REFERENCES

1 ICNARC. (2017). Key Statistics From The Case Mix Programme — Adult, General Critical Care Units, 2016-2017. Intensive Care National Audit and Research Centre: London.

2 Chan D, Reid T, White C et al. Influence of a regional centralised upper gastrointestinal cancer service model on patient safety, quality of care and survival. *Clin Oncol (R Coll Radiol)* 2015;25:719-25.

3 Myint PK, Lowe D, Stone RA, et al. National COPD Resources and Outcomes Project 2008: patients with chronic obstructive pulmonary disease exacerbations who present with radiological pneumonia have worse outcome compared to those with non-pneumonic chronic obstructive pulmonary disease. *Respiration* 2011;82:320–7.

4 Bridgewater B, Hickey GL, Cooper G, et al. Publishing cardiac surgery mortality rates: lessons for other specialties. *BMJ* 2013;346:f1139.

5 Department For Transport. Reported Road Casualties Great Britain: 2013 Annual Report. London: Department for Transport 2013; 1–11.

6 Daly K, Beale R, Chang RW. Reduction in mortality after inappropriate early discharge from intensive care unit: logistic regression triage model. *BMJ* 2001;322:1274–6.

7 Goldfrad C, Rowan K. Consequences of discharges from intensive care at night. *Lancet* 2000;355:1138–42.

8 Litton E, Ho KM, Lee KY et al. C-reactive protein concentration as a predictor of in-hospital mortality after ICU discharge: a nested case-control study. Intensive Care Med 2008;34:481–7.

9 NCEPOD. Knowing the Risk. A Review of the Peri-operative Care of Surgical Patients. London: National Confidential Enquiry into Patient Oucome and Death 2011.

10 NCEPOD. An acute problem. Nursing the elderly : in Hospital, Homes and the Community. London: National Confidential Enquiry into Patient Oucome and Death 2005..

11 NEPOD. Time to Intervene ? A Review of Patients who Underwent Cardiopulmonary Resuscitation as a Result of an In-hospital Cardiac Arrest. London: National Confidential Enquiry into Patient Oucome and Death 2012.

12 The Royal College of Surgeons. Emergency Surgery: Standards for unscheduled surgical care. London; 2011. Available from: https://www.rcseng.ac.uk/publications/docs/emergency-surgery-standards-for-unscheduled-care

13 Francis R. Report of the Mid Staffordshire NHS Foundation Trust Public Inquiry. London: The Stationary Office 2013. Available from: http://www.midstaffspublicinquiry.com/sites/default/files/report/Executive summary.pdf

14 Keogh B. Review into the quality of care and treatment provided by 14 hospital trusts in England: overview report. London: Hm Govt. 2013. Available from: http://www.nhs.uk/NHSEngland/bruce-keogh-review/Documents/outcomes/keogh-review-final-report.pdf

15 NCEPOD. Tracheostomy Care: On the Right Trach? London: National Confidential Enquiry into Patient Oucome and Death 2014.

16 Department of Health. Comprehensive Critical Care: A Review Of Adult Critical Care Services. London: Department of Health 2000.

For peer review only - http://bmjopen.bmj.com/site/about/guidelines.xhtml

17 Rowan K, Adam S, Ball C, et al. NIHR Service Delivery and Organisation programme. Evaluation of outreach services in critical care. Southampton; NIHR 2004.

18 Gao H, Harrison DA, Parry GJ, et al. The impact of the introduction of critical care outreach services in England: a multicentre interrupted time-series analysis. *Crit Care* 2007 11:R113.

19 Forsberg A, Lindgren E, Engström Å. Being transferred from an intensive care unit to a ward: Searching for the known in the unknown. *Int J Nurs Pract* 2011;17:110–6.

20 Leith BA. Patients' and family members' perceptions of transfer from intensive care. *Hear Lung J Acute Crit Care* 1999;28:210–8.

21 Ludin SM, Arbon P, Parker S. Patients' transition in the intensive care units: concept analysis. *Intensive Crit Care Nurs* 2013;29:187–92.

22 Strahan EHE, Brown RJ. A qualitative study of the experiences of patients following transfer from intensive care. *Intensive Crit Care Nurs* 2005;21:160–71.

23 Mckinney A, Deeny P. Leaving the intensive care unit: A phenomenological study of the patients' experience. *Intensive Crit Care Nurs* 2002;18:320–31.

24 Odell M. The patient's thoughts and feelings about their transfer from intensive care to the general ward. *J Adv Nurs* 2000 31:322–9.

25 Field K, Prinjha S, Rowan K. "One patient amongst many": a qualitative analysis of intensive care unit patients' experiences of transferring to the general ward. *Crit Care* 2008;12:R21.

26 Lin F, Chaboyer W, Wallis M, et al. Factors contributing to the process of intensive care patient discharge: an ethnographic study informed by activity theory. *Int J Nurs Stud* 2013;50:1054–66.

27 Häggström M, Asplund K, Kristiansen L. Struggle with a gap between intensive care units and general wards. *Int J Qual Stud Health Well-being* 2009;4:181–92.

28 Häggström M, Asplund K, Kristiansen L. How can nurses facilitate patient's transitions from intensive care?. A grounded theory of nursing. *Intensive Crit Care Nurs* 2012;28:224–33.

29 Whittaker J, Ball C. Discharge from intensive care: a view from the ward. *Intensive Crit Care Nurs* 2000;16:135–43.

30 Martinez GH, Fernandez R, Casado MS, et al. Tracheostomy tube in place at intensive care unit discharge is associated with increased ward mortality. *Respir Care* 2009;54:1644–52.

31 Fernandez R, Bacelar N, Hernandez G, et al. Ward mortality in patients discharged from the ICU with tracheostomy may depend on patient's vulnerability. *Intensive Care Med* 2008;34:1878–82.

32 Araújo I, Gonçalves-Pereira J, Teixeira S, et al. Assessment of risk factors for in-hospital mortality after intensive care unit discharge. *Biomarkers* 2012;17:180–5.

33 Ho KM, Lee KY, Dobb GJ, et al. C-reactive protein concentration as a predictor of in-hospital mortality after ICU discharge: A prospective cohort study. *Intensive Care Med* 2008;34:481–7.

34 Ranzani OT, Prada LF, Zampieri FG, et al. Failure to reduce C-reactive protein levels more than 25% in the last 24 hours before intensive care unit discharge predicts higher in-hospital mortality: A cohort study. *J Crit Care* 2012;27:525.e9-15.

35 Gantner D, Bailey M, Huckson S, et al. Mortality related to after-hours discharge from intensive care in Australia and New Zealand. *Intensive Care Med* 2014;40:1528–35.

36 Beck DH, McQuillan P, Smith GB. Waiting for the break of dawn? The effects of discharge time,

For peer review only - http://bmjopen.bmj.com/site/about/guidelines.xhtml

discharge TISS scores and discharge facility on hospital mortality after intensive care. *Intensive Care Med* 2002;28:1287–93.

37 Pilcher D V, Duke GJ, George C, et al. After-hours discharge from intensive care increases the risk of readmission and death. *Anaesth Intensive Care* 2007;35:477–85.

38 Laupland KB, Misset B, Souweine B, et al. Mortality associated with timing of admission to and discharge from ICU: a retrospective cohort study. *BMC Health Serv Res* 2011;11:321.

39 Priestap F, Martin CM. Impact of intensive care unit discharge time on patient outcome. *Crit Care Med* 2006;34:2946–51.

40 Uusaro A, Kari A, Ruokonen E. The effects of ICU admission and discharge times on mortality in Finland. *Intensive Care Med* 2003;29:2144–8.

41 Pilcher D, Duke GJ, George C, et al. Pilcher After-hours discharge from ICU. *Anaesth Intensive Care* 2007;35:477–85.

42 Vollam S, Dutton S, Lamb S, et al. Out-of-hours discharge from intensive care, in-hospital mortality and intensive care readmission rates: a systematic review and meta-analysis. *Intensive Care Medicine* 2018;44:1115-1129.

43 Lawrence A, Havill JH. An audit of deaths occurring in hospital after discharge from the intensive care unit. *Anaesth Intensive Care* 1999;27:185–9.

44 McLaughlin N, Leslie GD, Williams TA, et al. Examining the occurrence of adverse events within 72 hours of discharge from the intensive care unit. *Anaesth Intensive Care* 2007;35:486–93.

45 Mayr VD, Dünser MW, Greil V, et al. Causes of death and determinants of outcome in critically ill patients. *Crit Care* 2006;10:R154.

46 Trivedi M, Ridley SA. Intermediate outcome of medical patients after intensive care. *Anaesthesia* 2001;56(9):841–6.

47 Denehy L, Skinner EH, Edbrooke L, et al. Exercise rehabilitation for patients with critical illness: a randomized controlled trial with 12 months of follow-up. *Crit Care* 2013;17:R156.

48 Jackson JC, Ely EW, Morey MC, et al. Cognitive and physical rehabilitation of intensive care unit survivors: results of the RETURN randomized controlled pilot investigation. *Crit Care Med* 2012;40:1088–97.

49 Elliott D, McKinley S, Alison J, et al. Health-related quality of life and physical recovery after a critical illness: a multi-centre randomised controlled trial of a home-based physical rehabilitation program. *Crit Care* 2011;15:R142.

50 Adler J, Malone D. Early mobilization in the intensive care unit: a systematic review. Cardiopulm Phys Ther J 2012;23:5–13.

51 Walsh TS, Salisbury LG, Merriweather JL, et al. Increased hospital-based physical rehabilitation and information provision after intensive care unit discharge: The RECOVER randomized clinical trial. *JAMA Internal Medicine,* 2015;175,:901-910.

52 NHS National Quality Board. National Quality Board Patient Experience Framework. 2012; Available from: http://www.institute.nhs.uk/patient_experience/guide/the_policy_framework.html

53 Vincent C, Neale G, Woloshynowych M. Adverse events in British hospitals: preliminary retrospective record review. *BMJ* 2001;322:517–9.

For peer review only - http://bmjopen.bmj.com/site/about/guidelines.xhtml

54 NCEPOD. *Caring to the End? A Review of the Care of Patients Who Died Within Four Days of Hospital Admission.* London: National Confidential Enquiry into Patient Oucome and Death 2010.

55 Royal College of Physicians. National Mortality Case Record Review Programme: structured case note review data collection. London: Royal College of Physicians 2017.

56 Hogan H, Healey F, Neale G, et al. Preventable deaths due to problems in care in English acute hospitals: a retrospective case record review study. *BMJ Qual Saf* 2012;21:737–45.

57 Hutchinson A, Coster JE, Cooper KL, et al. A structured judgement method to enhance mortality case note review: development and evaluation. *BMJ Qual Saf*. 2013;22:1032–40.

58 Hogan H, Healey F, Neale G, et al. Learning from preventable deaths: exploring case record reviewers' narratives using change analysis. *J R Soc Med* 2014;107:365–75.

59 Odell M, Gerber K, Gager M. Activated Critical Care Outreach. *Methods* 2010;19:599-602.

60 Rance S, McCourt C, Rayment J, et al. Women's safety alerts in maternity care: is speaking up enough? *BMJ Qual Saf* 2013;22:348–55.

61 Ward JK, Armitage G. Can patients report patient safety incidents in a hospital setting? A systematic review. *BMJ Qual Saf* 2012;21:685–99.

62 Bryant A, Charmaz K. The SAGE Handbook of Grounded Theory. London: Sage Publications 2007.

63 Creswell J. Qualitative Inquiry And Research Design: Choosing Among Five Approaches. London: Sage Publications 2012.

64 Pope C, Ziebland S, Mays N. Qualitative research in health care. Analysing qualitative data. *BMJ* 2000;320:114–6.

65 Coyne IT. Sampling in qualitative research. Purposeful and theoretical sampling; merging or clear boundaries? *J Adv Nurs* 1997;26:623–30.

66 Parkes CM. Guidelines for conducting ethical bereavement research. *Death Stud* 1995;19:171–81.

67 Bentley B, O'Connor M. Conducting research interviews with bereaved family carers: when do we ask? *J Palliat Med* 2014;18:241–5.

68 Chapple A, Ziebland S, Hawton K. Taboo and the different death? Perceptions of those bereaved by suicide or other traumatic death. *Sociol Heal Illn* 2015;37:610–25.

69 Walker D, Myrick F. Grounded theory: an exploration of process and procedure. *Qual Health Res* 2006;16:547–59.

70 Hinton L, Locock L, Knight M. Maternal critical care: what can we learn from patient experience? A qualitative study. *BMJ Open* 2015;5:e006676.

71 Ziebland S, McPherson A. Making sense of qualitative data analysis: an introduction with illustrations from DIPEx (personal experiences of health and illness). Med Educ. 2006;40(5):405–14.

72 Medical Research Council. A framework for development and evaluation of RCTs for complex interventions to improve health. London; MRC 2000.

Chan A-W, Tetzlaff JM, Altman DG, et al. SPIRIT 2013 Statement: Defining standard protocol items for clinical trials. Ann Intern Med 2013;158:200-207.

For peer review only

For peer review only - http://bmjopen.bmj.com/site/about/guidelines.xhtml

FIGURE LEGENDS:

Figure 1. Primary data collection

Figure 2. Modelling the intervention

For peer review only

For peer review only - http://bmjopen.bmj.com/site/about/guidelines.xhtml

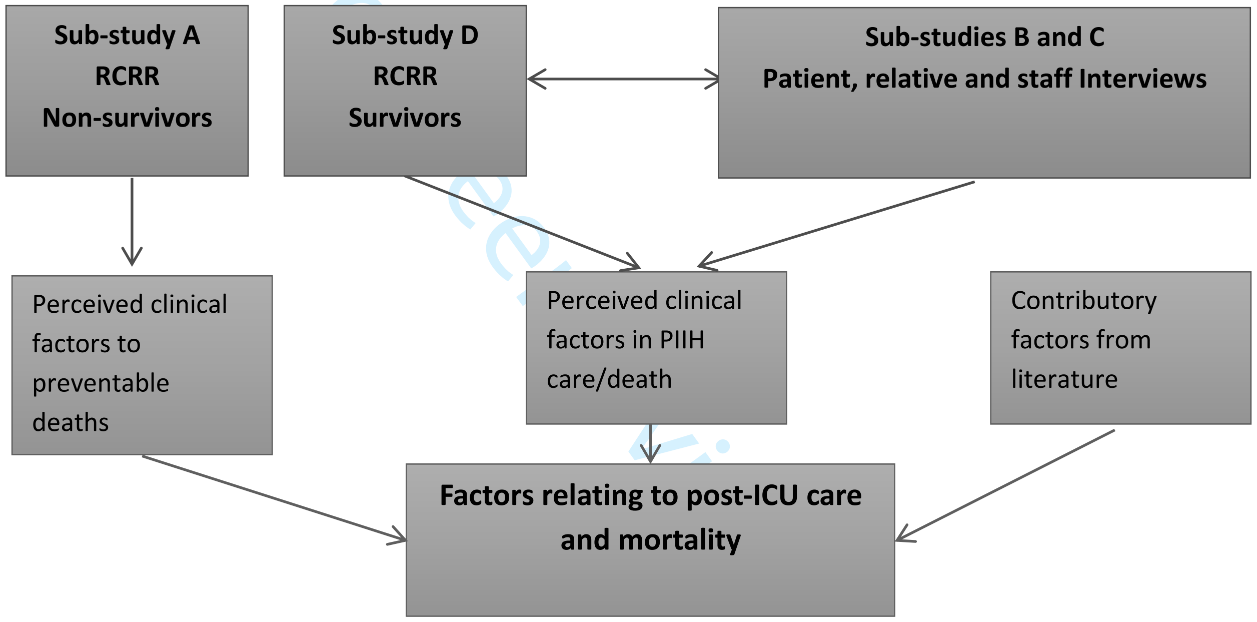

Figure 1. Work flow

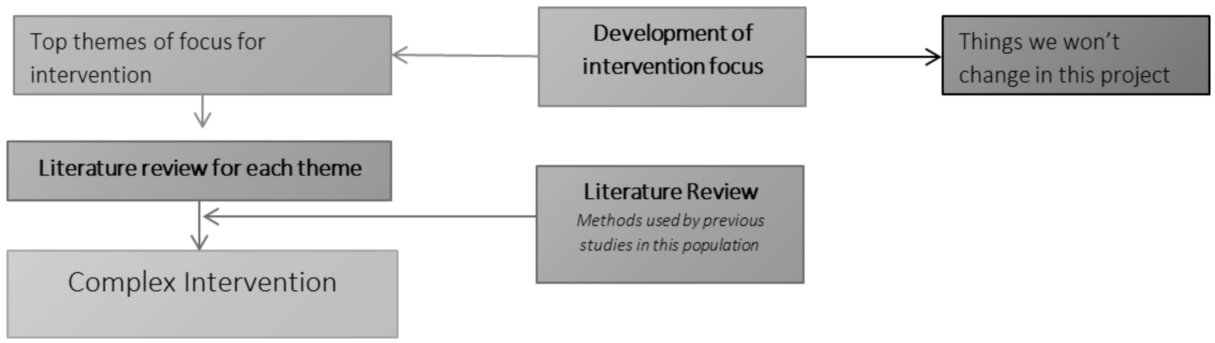

For peer review only

SPIRIT — STANDARD PROTOCOL ITEMS: RECOMMENDATIONS FOR INTERVENTIONAL TRIALS

SPIRIT 2013 Checklist: Recommended items to address in a clinical trial protocol and related documents*

Section/item	ItemNo	Description	
**Administrative information**			
Title	1	Descriptive title identifying the study design, population, interventions, and, if applicable, trial acronym	p1
Trial registration	2a	Trial identifier and registry name. If not yet registered, name of intended registry	Abstract and p 9
	2b	All items from the World Health Organization Trial Registration Data Set	As per registry
Protocol version	3	Date and version identifier	p9
Funding	4	Sources and types of financial, material, and other support	p10
Roles and responsibilities	5a	Names, affiliations, and roles of protocol contributors	p1
	5b	Name and contact information for the trial sponsor	p9
	5c	Role of study sponsor and funders, if any, in study design; collection, management, analysis, and interpretation of data; writing of the report; and the decision to submit the report for publication, including whether they will have ultimate authority over any of these activities	n/a
	5d	Composition, roles, and responsibilities of the coordinating centre, steering committee, endpoint adjudication committee, data management team, and other individuals or groups overseeing the trial, if applicable (see Item 21a for data monitoring committee)	p3
**Introduction**			
Background and rationale	6a	Description of research question and justification for undertaking the trial, including summary of relevant studies (published and unpublished) examining benefits and harms for each intervention	p3
	6b	Explanation for choice of comparators	n/a
Objectives	7	Specific objectives or hypotheses	p4
Trial design	8	Description of trial design including type of trial (eg, parallel group, crossover, factorial, single group), allocation ratio, and framework (eg, superiority, equivalence, noninferiority, exploratory)	p4

For peer review only

Methods: Participants, interventions, and outcomes

Study setting	9	Description of study settings (eg, community clinic, academic hospital) and list of countries where data will be collected. Reference to where list of study sites can be obtained	p5
Eligibility criteria	10	Inclusion and exclusion criteria for participants. If applicable, eligibility criteria for study centres and individuals who will perform the interventions (eg, surgeons, psychotherapists)	P4-5
Interventions	11a	Interventions for each group with sufficient detail to allow replication, including how and when they will be administered	For interviews: p5
	11b	Criteria for discontinuing or modifying allocated interventions for a given trial participant (eg, drug dose change in response to harms, participant request, or improving/worsening disease)	n/a
	11c	Strategies to improve adherence to intervention protocols, and any procedures for monitoring adherence (eg, drug tablet return, laboratory tests)	n/a
	11d	Relevant concomitant care and interventions that are permitted or prohibited during the trial	n/a
Outcomes	12	Primary, secondary, and other outcomes, including the specific measurement variable (eg, systolic blood pressure), analysis metric (eg, change from baseline, final value, time to event), method of aggregation (eg, median, proportion), and time point for each outcome. Explanation of the clinical relevance of chosen efficacy and harm outcomes is strongly recommended	p8
Participant timeline	13	Time schedule of enrolment, interventions (including any run-ins and washouts), assessments, and visits for participants. A schematic diagram is highly recommended (see Figure)	n/a
Sample size	14	Estimated number of participants needed to achieve study objectives and how it was determined, including clinical and statistical assumptions supporting any sample size calculations	p7
Recruitment	15	Strategies for achieving adequate participant enrolment to reach target sample size	p6

Methods: Assignment of interventions (for controlled trials)

Allocation:

Sequence generation	16a	Method of generating the allocation sequence (eg, computer-generated random numbers), and list of any factors for stratification. To reduce predictability of a random sequence, details of any planned restriction (eg, blocking) should be provided in a separate document that is unavailable to those who enrol participants or assign interventions	n/a
Allocation concealment mechanism	16b	Mechanism of implementing the allocation sequence (eg, central telephone; sequentially numbered, opaque, sealed envelopes), describing any steps to conceal the sequence until interventions are assigned	n/a
Implementation	16c	Who will generate the allocation sequence, who will enrol participants, and who will assign participants to interventions	n/a
Blinding (masking)	17a	Who will be blinded after assignment to interventions (eg, trial participants, care providers, outcome assessors, data analysts), and how	n/a
	17b	If blinded, circumstances under which unblinding is permissible, and procedure for revealing a participant's allocated intervention during the trial	n/a
**Methods: Data collection, management, and analysis**			
Data collection methods	18a	Plans for assessment and collection of outcome, baseline, and other trial data, including any related processes to promote data quality (eg, duplicate measurements, training of assessors) and a description of study instruments (eg, questionnaires, laboratory tests) along with their reliability and validity, if known. Reference to where data collectionforms can be found, if not in the protocol	p4-5
	18b	Plans to promote participant retention and complete follow-up, including list of any outcome data to be collected for participants who discontinue or deviate from intervention protocols	n/a
Data management	19	Plans for data entry, coding, security, and storage, including any related processes to promote data quality (eg, double data entry; range checks for data values). Reference to where details of data management procedures can be found, if not in the protocol	p7
Statistical methods	20a	Statistical methods for analysing primary and secondary outcomes. Reference to where other details of the statistical analysis plan can be found, if not in the protocol	p7-8
	20b	Methods for any additional analyses (eg, subgroup and adjusted analyses)	p7-8

For peer review only - http://bmjopen.bmj.com/site/about/guidelines.xhtml

	20c	Definition of analysis population relating to protocol non-adherence (eg, as randomised analysis), and any statistical methods to handle missing data (eg, multiple imputation)	n/a
**Methods: Monitoring**			
Data monitoring	21a	Composition of data monitoring committee (DMC); summary of its role and reporting structure; statement of whether it is independent from the sponsor and competing interests; and reference to where further details about its charter can be found, if not in the protocol. Alternatively, an explanation of why a DMC is not neede	n/a
	21b	Description of any interim analyses and stopping guidelines, including who will have access to these interim results and make the final decision to terminate the trial	n/a
Harms	22	Plans for collecting, assessing, reporting, and managing solicited and spontaneously reported adverse events and other unintended effects of trial interventions or trial conduct	p9
Auditing	23	Frequency and procedures for auditing trial conduct, if any, and whether the process will be independent from investigators and the sponsor	n/a
**Ethics and dissemination**			
Research ethics approval	24	Plans for seeking research ethics committee/institutional review board (REC/IRB) approval	p9
Protocol amendments	25	Plans for communicating important protocol modifications (eg, changes to eligibility criteria, outcomes, analyses) to relevant parties (eg, investigators, REC/IRBs, trial participants, trial registries, journals, regulators)	n/a
Consent or assent	26a	Who will obtain informed consent or assent from potential trial participants or authorised surrogates, and how (see Item 32)	p7
	26b	Additional consent provisions for collection and use of participant data and biological specimens in ancillary studies, if applicable	n/a
Confidentiality	27	How personal information about potential and enrolled participants will be collected, shared, and maintained in order to protect confidentiality before, during, and after the trial	p7
Declaration of interests	28	Financial and other competing interests for principal investigators for the overall trial and each study site	p10
Access to data	29	Statement of who will have access to the final trial dataset, and disclosure of contractual agreements that limit such access for investigators	n/a

For peer review only

Ancillary and post-trial care	30	Provisions, if any, for ancillary and post-trial care, and for compensation to those who suffer harm from trial participation	p9-10
Dissemination policy	31a	Plans for investigators and sponsor to communicate trial results to participants, healthcare professionals, the public, and other relevant groups (eg, via publication, reporting in results databases, or other data sharing arrangements), including any publication restrictions	p10
	31b	Authorship eligibility guidelines and any intended use of professional writers	p10
	31c	Plans, if any, for granting public access to the full protocol, participant-level dataset, and statistical code	n/a
**Appendices**			
Informed consent materials	32	Model consent form and other related documentation given to participants and authorised surrogates	p7 (on trial registry)
Biological specimens	33	Plans for collection, laboratory evaluation, and storage of biological specimens for genetic or molecular analysis in the current trial and for future use in ancillary studies, if applicable	n/a

*It is strongly recommended that this checklist be read in conjunction with the SPIRIT 2013 Explanation & Elaboration for important clarification on the items. Amendments to the protocol should be tracked and dated. The SPIRIT checklist is copyrighted by the SPIRIT Group under the Creative Commons "Attribution-NonCommercial-NoDerivs 3.0 Unported" license.

For peer review only - http://bmjopen.bmj.com/site/about/guidelines.xhtml

BMJ Open

Protocol for a mixed methods exploratory investigation of care following intensive care discharge: the REFLECT study.

Journal:	*BMJ Open*
Manuscript ID	bmjopen-2018-027838.R1
Article Type:	Protocol
Date Submitted by the Author:	19-Nov-2018
Complete List of Authors:	Vollam, Sarah; University of Oxford, Nuffield Department of Neurosciences<br>Gustafson, Owen; Oxford University Hospitals NHS Foundation Trust, Adult Intensive Care Unit<br>Hinton, Lisa; University of Oxford, Health Experiences Research Group, Nuffield Department of Primary Care Health Sciencs<br>Morgan, Lauren; University of Oxford, Nuffield Department of Surgical Sciences<br>Pattison, Natalie; University of Hertfordshire, School of Health and Social Work<br>Thomas, Hilary; University of Hertfordshire, CRIPACC<br>Young, Duncan; Oxford University, Nuffield Department of Clinical Neurosciences<br>Watkinson, Peter; University of Oxford, Nuffield Department of Clinical Neurosciences
**Primary Subject Heading**:	Intensive care
Secondary Subject Heading:	Health services research
Keywords:	Mixed methods, Critical Care, Outcome, Protocol

SCHOLARONE™
Manuscripts

Protocol for a mixed methods exploratory investigation of care following intensive care discharge: the REFLECT study.

Sarah Vollam (Corresponding author)
Nuffield Department of Clinical Neurosciences
University of Oxford
Level 6, West Wing, John Radcliffe Hospital
Oxford
OX3 9DU
UK
Sarah.vollam@ndcn.ox.ac.uk
01865 231440

Owen Gustafson
Adult Intensive Care Unit
Oxford University Hospitals NHS Foundation Trust
Oxford
UK

Dr Lisa Hinton
Nuffield Department of Primary Health Care
University of Oxford
Oxford
UK

Dr Lauren Morgan
Nuffield Department of Surgical Sciences
University of Oxford
Oxford
UK

Prof Natalie Pattison
School of Health and Social Work
University of Hertfordshire
Hatfield
UK

Prof Hilary Thomas
Centre for Research in Public Health and Community Care
University of Hertfordshire
Hatfield
UK

J. Duncan Young
Nuffield Department of Clinical Neurosciences
University of Oxford
Oxford
UK

Assoc Prof Peter Watkinson
Nuffield Department of Clinical Neurosciences
University of Oxford
Oxford
UK

Word count: 3,954

For peer review only

For peer review only - http://bmjopen.bmj.com/site/about/guidelines.xhtml

ABSTRACT

Introduction

A substantial number of patients discharged from Intensive Care Units (ICUs) subsequently die without leaving hospital. It is unclear how many of these deaths are preventable. Ward-based management following discharge from ICU is an area that patients and healthcare staff are concerned about. The primary aim of REFLECT (Recovery Following Intensive Care Treatment) is to develop an intervention plan to reduce in-hospital mortality rates in patients who have been discharged from ICU.

Methods and analysis

REFLECT is a multicentre mixed methods exploratory study examining ward care delivery to adult patients discharged from ICU. The study will be made up of four sub-studies. Medical notes of patients who were discharged from intensive care and subsequently died will be examined using a Retrospective Case Records Review (RCRR) technique. Patients and their relatives will be interviewed about their post-ICU care, including relatives of patients who died in hospital following ICU discharge. Staff involved in the care of patients post-ICU discharge will be interviewed about the care of this patient group. The medical records of patients who survived their post-ICU stay will also be reviewed using the RCRR technique. The analyses of the sub-studies will be both descriptive and use a modified grounded theory approach to identify emerging themes. The evidence generated in these four sub-studies will form the basis of the intervention development, which will take place through stakeholder and clinical expert meetings.

Ethics and dissemination

Ethical approval has been obtained through the Wales Research and Ethics Committee 4 (17/WA/0107). We aim to disseminate the findings through international conferences, international peer-reviewed journals and social media.

Trial registration number

ISRCTN14658054

ARTICLE SUMMARY

Strengths and limitations of this study

- This exploratory study uses mixed methods to gather rich data from multiple perspectives to inform the development of an intervention.
- This protocol has been designed using MRC guidance on the development of complex interventions.
- As this is a complex cohort of patients, it is not clear whether problems in care will be distinct enough to be amenable to change through an intervention.

For peer review only

INTRODUCTION

In 2015-16, over 8000 of the 134,000 patients discharged from Intensive Care Units (ICUs) in England and Wales died without leaving hospital.[1] This mortality rate is higher than hospitalised groups considered to be at high risk [2–4] and is more than five times the annual number of UK road traffic accident deaths.[5]

Most patients discharged from ICU are expected to go home ([6] and preliminary analysis provided by Intensive Care National Audit and Research Centre. There are widely varying in-hospital post-ICU mortality rates (2.9% to 22.6%) for patients of similar illness severity at admission to ICU.[7,8] Several studies of general ward populations indicate changes in care could lead to improvements in outcome.[9–15]

In 2000, the Department of Health (DH) recognised the need to improve outcomes in this vulnerable patient group, recommending the introduction of critical care outreach "to support the continuing recovery of discharged patients on wards…".[16] The DH provided substantial financial support to establish these teams. The teams are costly, often constituted of skilled senior critical care practitioners.[17] However, there is limited evidence in terms of outreach efficacy on reducing mortality in the post-ICU population.[18]

Qualitative studies with patients [19–25] and staff [26–29] have identified problems with the transition from ICU to ward care. Many have focused on the psychological impact rather than clinical care, although one study found patients were concerned about the quality and availability of nursing and medical care on the wards.[25] A secondary analysis these interviews conducted by the Health Experience Research Group was undertaken as preparatory work for this study (http://www.healthtalk.org). We found patients were able to identify problems in care delivery such as lack of specific clinical skills and awareness of level of physical dependency.

Some studies have investigated which patients are most at risk. Potentially modifiable risk factors identified at ICU discharge include the presence of tracheostomy, [30–32] elevated C-reactive protein [8,33-34] or creatinine [34] and most compellingly, discharge out of hours [7,35–42]. The evidence identifying risk factors present on the ward after ICU discharge is currently somewhat limited.[43–46] There have been several single intervention, physical therapy-based strategies which alone have not been found to improve mortality.[47–50] Recently the RECOVER study reported no effect from delivering increased physiotherapy and dietetic advice to hospitalised patients following ICU discharge.[51] The history of interventions tried in this patient group emphasises the need to carefully establish an appropriate intervention package to trial. There is currently insufficient information about the ward management of these patients to know what an effective intervention aimed at reducing post-ICU in-hospital mortality would contain. Recent NHS guidance [52] has emphasised the need to incorporate patient experiences to improve their care. In combination with the experience of the carers in the ward environment, evidence from patients provides the most immediate information on identifiable problems with the care they receive. Additionally, case review has previously been shown to yield valuable information with which to improve ward-based care.[9,10,53-54]

The problem is urgent. Over 8,000 patients died in 2017 in hospital following discharge from intensive care. It is not currently known what proportion of these are expected deaths, but a substantial proportion of these deaths may be avoidable. The operation of ICU outreach teams throughout the country would greatly benefit from the development of an evidence-based care package.

For peer review only - http://bmjopen.bmj.com/site/about/guidelines.xhtml

METHODS

Objectives

Our primary objective is to develop a multifaceted human factors-based intervention to reduce in-hospital mortality rates in patients who have been discharged from intensive care. Our secondary objectives are to identify examples of high-quality care and areas for improvement.

Patient and Public Involvement

A patient and public (PPI) focus group was conducted during development of this study. The group were consulted on the design of the study with focus on patient/relative interviews approach and the burden of participating. Two members of this group are members of the steering committee. They have been consulted on the ongoing conduct on the study and have provided feedback on participant documentation.

General design

REFLECT is a multicentre mixed methods exploratory study examining ward care delivery to patients discharged from intensive care. Data collection is split into four sub-studies: a retrospective case records review (RCRR) of deceased patients, patient and relative interviews/focus groups, staff interviews/focus groups, and a RCRR of survivors (Figure 1).

RCRR deceased patients

Medical notes of patients who were transferred to wards from ICU and subsequently died will be examined using a Retrospective Case Record Review (RCRR) technique. This review will use an adaptation of a validated tool for making safety and quality judgements about care delivery.[55–57] Medical notes are reviewed and 'structured judgement' statements are made about the delivery of care. These statements are explicit, value-based comments on care delivery. The output of this is a relatively short but rich account of care delivery, identifying both good and poor care. The output of this stage will be a collation of care delivery, both where it has been excellent and where improvements could be made. This approach has been used extensively in other patient groups,[53,56] but not previously in this population. It is currently being adopted by the DH as a clinical governance tool within trusts as the National Mortality Care Record Review Programme.[55] It contains guidance to ensure a consistent and valid approach. We have piloted this review methodology and undertaken preparatory work to ensure the methodology will capture where novel processes could change outcomes for hospitalised patients discharged from Intensive Care. Training will be conducted with the three researchers involved in these reviews, to ensure consistency of findings.

Cases where differences in care delivery could improve outcomes will be further analysed using the 'change analysis' method developed by Hogan et al.[58] This is an in-depth qualitative analysis of the narrative account of care delivery for each patient, using a human factors framework. The analysis will allow identification of areas where novel care processes could change outcomes, and what processes could facilitate this. These findings will guide the design and implementation of the intervention.

Patient and relative interviews/focus groups

Patients and their relatives are ideally placed to offer reflection and critique of their care.[59–61] Our secondary analysis of relative and patient interviews showed patients and relatives could clearly

For peer review only - http://bmjopen.bmj.com/site/about/guidelines.xhtml

identify areas of their post-ICU ward care which they considered unsatisfactory. However, discussions about post-ICU care were limited as the interviews spanned the entire hospital experience. Further interviews with survivors and their relatives are required to focus on how care on the wards following ICU discharge could be improved. Focus groups will be offered where more than three people are interested in participating on a given day. Telephone interviews will also be offered as an alternative to face-to-face interview.

We will also interview relatives of patients who died in hospital following intensive care discharge, to ensure that their experiences are included (involving relatives of patients who died was recommended by our patient and public involvement (PPI) group). This will provide a unique perspective and augment the findings of the RCRR of deceased patients. A focus group or telephone option will not be offered to this group due to the potential for the participant to become distressed, as this would not allow appropriate management of the interview.

Staff interviews/focus groups

We will conduct interviews with staff, with focus groups offered where more than three staff members are able to attend together. Interviews/focus groups will be conducted with a variety of staff members to encourage a multi-disciplinary analysis of this area of care. Telephone interviews will be offered as an alternative to face to face interviews.

Interviews with patients and staff will be conducted in parallel so that emerging themes can be explored across groups. The interviews will build on themes identified in the preliminary secondary analysis and evidence synthesis discussed above. This work will take an approach informed by the tenets of grounded theory, reflecting the inductive approach to developing an understanding of this area of care.[62-63] Interviews and focus groups will use a topic guide, based on completed work and input from patient representatives. We anticipate the topic guide will evolve throughout the interviews/focus group phase to ensure any emerging themes are explored,[64] reflecting the iterative nature of qualitative research.

RCRR survivors

We will review the case records of patients who survived their post-ICU ward stay. Ideally, all patients who were interviewed will be included (subject to participant consent). The reviews will follow the same structure proposed for reviewing deceased patient medical notes. This will be modified to assess examples of high quality care and areas for improvement (using structured judgement and clear rationale). All cases will be further analysed using the 'change analysis' method described above. We will triangulate areas identified by patients and relatives with those found in the case records and compare with those identified for non-survivors.

Study setting

The study is taking place in three separate United Kingdom NHS Trusts. There are approximately 2000 patients discharged from the general adult ICUs across the three trusts annually. The RCRR and patient, relative and staff interviews will occur at all three trusts. The specialist cardiothoracic and neurosurgical ICUs will not be included in the study.

Participant selection

RCRR deceased patients

For peer review only - http://bmjopen.bmj.com/site/about/guidelines.xhtml

Patients will be identified by a search of the local NHS database. The most recent 300 patients who were discharged from ICU and died during the same hospital admission will be identified and their medical records retrieved. All patients aged 18 years or above discharged from ICU to a ward who died prior to hospital discharge will be included. Any patients with inaccessible medical notes will be excluded.

Patient and relative interviews/focus groups

Patients discharged from hospital: patients invited to attend the intensive care follow-up clinic will also be invited to participate in semi-structured interviews. Their relatives will also be invited and may participate either as well as or instead of the patient. This invitation will be issued by the clinic organiser (a member of the direct care team). Patients will be eligible if they are willing and able to give informed consent, are 18 years or older and are a patient or relative of a patient who was discharged from ICU to a ward and survived to hospital discharge. Patients will be excluded if they lack the capacity to consent or have poor spoken English as it will not be possible to conduct the interviews through an interpreter. Participants will be sought with varying experiences, to facilitate maximum variation in the sample.[65]

Patients who did not survive to hospital discharge: our planned involvement of relatives of patients who died follows advice from two experts in the field, Dr Colin Parkes (emeritus Senior Lecturer in Psychiatry, Royal London Hospital) and Prof Maggie Stroebe-Harrold (University of Utrecht), published guidelines,[66] bereavement research,[67] and advice from the study PPI group. A pack will be sent by the ICU follow-up team to relatives of patients who were discharged from ICU and subsequently died on a ward. This will include a covering letter, brief leaflet and Participant Information Sheet. Letters will be sent out 6 months following the relative's death, as suggested by bereavement research.[66-67] The letter will invite the relative to consider the study and contact the study team if they are interested. It will clearly state that they are very welcome to completely discard the letter and no further contact will be made. It will also be made clear that if they do participate, they can withdraw at any time, including during the interview.

If we are unable to recruit participants through this approach we may contact local support groups, such as ICUSteps (www.icusteps.org) to explore recruitment through them. The study has been endorsed by the national ICUSteps group. In this instance, packs (including covering letter, leaflet and PIS) would be given out by the group facilitator if, and when, they felt this was appropriate. This direct approach is used successfully by the Health Experience Research Group in many of their studies, including those recruiting bereaved relatives.[25,68] Participants will be included if they are willing and able to give informed consent, are 18 years or older and are a relative of a patient who was discharged from ICU and did not survive to hospital discharge. As with survivor interviews, participants will be excluded if they lack the capacity to consent or have poor spoken English.

Staff interviews/focus groups

Staff involved in the care of patients discharged from ICU to the wards (including nurses, doctors, physiotherapists, dieticians and other allied health professionals) will be recruited to participate in interviews/focus groups. As above, purposive sampling will be utilised to ensure a diverse range of exposure, experience and background training. Invitation letters and attached participant information sheets will be distributed to all staff by ward clerks, or a similar member of staff to wards with a high throughput of post-ICU patients. In addition: posters will be placed on wards, advertisements placed on trust-wide intranet and prior contact with senior managers will be sought for endorsement. We also anticipate an element of snowballing from other participants. Participants

For peer review only - http://bmjopen.bmj.com/site/about/guidelines.xhtml

will be included if they are willing and able to give informed consents, are aged 18 years or older and are a member of NHS staff involved in the care of patients discharged from ICU to the wards. There are no exclusion criteria.

RCRR survivors

Patients who are approached to participate in the interview study will also be asked to participate in the RCRR. Ideally, all those who are interviewed will consent to notes review, but it is anticipated that some may not. Patients may consent to the RCRR without participating in the interview study. Information about the study will be sent out with the ICU follow-up clinic appointment, around two weeks in advance. Participants will be included if they are willing and able to give informed consent, are aged 18 years or older and have been discharged from ICU to the ward and subsequently discharged from hospital.

Consent

Consent will not be obtained for the RCRR for deceased patients. Support to access notes for this group will be sought from the Confidentiality Advisory Group, who advise the Health Research Authority on applications to process patient information without consent. For patients/relatives undertaking interviews, consent will be sought by trained researchers at the time of interview if face-to-face. Postal consent will be offered as an alternative if the participant requests a telephone interview or for notes review only. If the patient opts for notes review only, they may sign and return the consent form without speaking with the research team. The patient will be able to discuss the study with a member of the study team prior to signing the consent form if they wish. Documents relating to informed consent are available within the trial registry.

Sample size

RCRR deceased patients: based on previous audit, up to 300 patient records will be reviewed, yielding approximately 30 records for in-depth analysis. These records will be sourced from all three trusts.

Patient and relative interviews: we estimate approximately 20 interviews will be required to supplement data from our secondary analysis of patient and relative interviews. We anticipate these participants will be recruited from all three trusts. Data collection will continue with concurrent thematic analysis, until theoretical saturation has been reached (i.e. no new themes are emerging). Anticipated numbers are given for each group, but may vary to achieve saturation.[62-63]

Staff interviews: we anticipate conducting interviews/focus groups with approximately 30 staff members, across all three trusts.

RCRR survivors: up to 30 patient records (to match the number for in-depth analysis above). We anticipate these will be recruited from across the three trusts.

Data storage

All electronic data will be password-protected and stored on a secure server within a university research facility. All paper documentation (such as consent forms and case report forms) will be stored in a locked university research facility behind two swipe access doors.

Data analysis

RCRR deceased and survivors

For peer review only - http://bmjopen.bmj.com/site/about/guidelines.xhtml

Statistical analysis will be mostly descriptive. This will include proportions of patients experiencing one or more 'problem with care'. For deceased patients, we will report the proportion of cases deemed to have more than a 50% chance of death being avoidable. Avoidability will be judged based on the case record review and decisions discussed and verified between the three researchers conducting the RCRR. For survivors we will report proportion of cases who experienced examples of high-quality care and areas where improvements could be made. Cases where improvements could be made (perhaps using examples of high quality care) will be further analysed using the 'change analysis' method developed by Hogan et al.[58] This additional analysis will add an in-depth qualitative analysis of the links between identified 'care areas' and associated human factors. This is particularly useful in cases with multiple complex problems, anticipated to be the case in this population.

We will triangulate "care areas" identified by patients and relatives with those found in the case records. We will compare the 'care areas' identified with those identified for non-survivors. Records will be reviewed after interview, to avoid any potential conflict of interest for the researcher.

A report will be produced summarising the potential areas and approaches for interventions and the human factors which contributed to the identified "care areas".

Interviews and focus groups

Audio recordings will be transcribed verbatim and entered into qualitative analysis software (NVivo). Interviews and focus groups will be transcribed verbatim into a specialist software package for coding qualitative data (QSR NVivo). A modified grounded theory approach will be used to identify emerging themes. This will ensure identification of "care areas" important to patients and health professionals, as well as those that researchers anticipate.[62-63,69] This approach has previously been used to identify areas of care which patients believed could be improved.[25,70-71]

Preliminary coding will take place soon after the interviews are conducted. This will allow any emerging themes to be explored in subsequent interviews. Preliminary coding will be refined using the method of constant comparison (until no new themes emerge) to produce a report for each theme.[62] Each report will reflect the most important themes that participants talk about in their interviews and represent the full range of experiences included in the interviews. These reports will reviewed and themes will be verified within the research team, comprising of four qualitative researchers (SV, HT, NP and LH).[71] Any differences in interpretation or emphasis will be discussed and resolved. For the final output, these themes will be further categorised by areas of care which could be improved, and suggestions for improvement.

Modelling the Intervention

Stakeholder meeting

The evidence generated through the methodology above, will form the basis of the intervention development (Figure 2). Guided by a Human Factors researcher, a stakeholder group will prioritise areas for intervention from those identified in the interviews, focus groups, case record reviews and our earlier research. The meeting will take the form of a prioritisation exercise, including a facilitated card sort to rank the potential areas for improvement. They will select the most promising areas that can be pragmatically combined in a multi-faceted intervention. For an area to be prioritised, the mechanism by which intervention in that area could be expected to reduce mortality will need to be defined.

For peer review only - http://bmjopen.bmj.com/site/about/guidelines.xhtml

Literature searches

We will then undertake literature searches to check if our prioritised areas have been previously investigated in other hospitalised patient populations. To capture relevant successful methods for change implementation we will review previous implementation methods for interventions in the post-ICU hospitalised patient group and methods used in studies of our prioritised areas in other hospitalised patient populations. This will result in a refined list of areas for inclusion and identification of previous methods used to successfully implement change in these areas.

Paper modelling exercise

Components of the multi-faceted intervention will be examined in an initial paper modelling exercise.[72] This exercise will allow exploration of: the interdependencies of the components, different implementation strategies and challenges that may be encountered.

Clinical experts meeting

The prioritised areas and the results of the paper modelling exercise will be taken to meeting of stakeholders and clinical experts. At this meeting the proposed intervention will be finalised with input from those likely to deliver the intervention and those who have previously experienced care.

ETHICS AND DISSEMINATION

Ethics

The study has received ethical approval from the Wales Research Ethics Committee (Ref: 17/WA/0107). The University of Oxford will act as sponsor. The study will be overseen by a steering committee and includes PPI involvement throughout.

This trial is registered: ISRCTN14658054. This paper reports protocol version 1 (April 2017), and has been written with reference to the SPIRIT checklist.[73]

RCRR deceased patients

As informed consent cannot be obtained for deceased patients in this sub-study, an application has been approved by the Confidentiality Advisory Group for suspension of the duty of confidentiality under section 251 of the NHS Act 2006 specifically in relation to this section of the project. The research brings the possibility of identification of areas where practice may not have been optimal, which will be referred through the organisations standard clinical governance processes. The response will follow the guidance given by the Royal College of Physicians Clinical governance guide to mortality case record reviews.[55]

Patient and relative interviews

Where possible, for patients, these interviews/focus groups will take place on the same day as their ICU follow up clinic appointment, where support will be available should the interview raise issues that may cause distress. For patients and relatives requiring further support appropriate referrals will be made within the existing hospital system and details of organisations outside the hospital offered.

Relatives of deceased patients will be identified and sensitively approached as discussed above. Training on talking with bereaved relatives will be provided for researchers. We will also use the

For peer review only - http://bmjopen.bmj.com/site/about/guidelines.xhtml

For peer review only

'buddy' system utilised by the Health Experiences Research Group, whereby another researcher will be available to debrief after each interview if necessary.

Staff interviews/focus groups

Given the sensitive nature of this subject, it is possible that discussions may cause distress to staff members. NHS Trust Occupational Health will be made aware that we are conducting this study and any staff member who causes concern to the researchers will be signposted to occupational health in the first instance.

Any answers which cause concern in terms of professional conduct will be discussed with clinicians within their management structure in the first instance, with a view to raising this with the line manager of the participant. Any disclosures raising serious concerns about a specific patient will be dealt with as described above.

RCRR survivors

It is anticipated that most patients participating in the RCRR will also be interviewed. In order to ensure there is no bias or conflict of interest which might influence the conversation, these reviews will be completed after the interviews. Any identified significant care areas will be escalated as outlined for the RCRR for deceased patients.

Dissemination

Results from this study will be disseminated at regional and international conferences and in peer-reviewed journals. Authorship of any papers related to this study will follow the ICMJE recommendations (http://www.icmje.org/recommendations/).

Data sharing

Consent was given by participants for anonymised data to be made available to other researchers undertaking relevant research. Applications to use anonymised data will be considered by the steering committee.

CONTRIBUTORS

SV, PW and DY conceived the project. SV, PW, DY, HT, LM and LH developed the protocol. OG drafted the manuscript. PW, NP and HT are providing PhD supervision for SV and supporting data analysis. All authors contributed to and revised the final manuscript.

FUNDING STATEMENT

This paper presents independent research funded by the National Institute for Health Research (NIHR) under its Research for Patient Benefit (RfPB) Programme (Grant Reference Number PB-PG-0215-36149). The views expressed are those of the author(s) and not necessarily those of the NHS, the NIHR or the Department of Health.

COMPETING INTERESTS

None declared.

For peer review only - http://bmjopen.bmj.com/site/about/guidelines.xhtml

REFERENCES

1 ICNARC. (2017). Key Statistics From The Case Mix Programme — Adult, General Critical Care Units, 2016-2017. Intensive Care National Audit and Research Centre: London.

2 Chan D, Reid T, White C et al. Influence of a regional centralised upper gastrointestinal cancer service model on patient safety, quality of care and survival. *Clin Oncol (R Coll Radiol)* 2015;25:719-25.

3 Myint PK, Lowe D, Stone RA, et al. National COPD Resources and Outcomes Project 2008: patients with chronic obstructive pulmonary disease exacerbations who present with radiological pneumonia have worse outcome compared to those with non-pneumonic chronic obstructive pulmonary disease. *Respiration* 2011;82:320–7.

4 Bridgewater B, Hickey GL, Cooper G, et al. Publishing cardiac surgery mortality rates: lessons for other specialties. *BMJ* 2013;346:f1139.

5 Department For Transport. Reported Road Casualties Great Britain: 2013 Annual Report. London: Department for Transport 2013; 1–11.

6 Daly K, Beale R, Chang RW. Reduction in mortality after inappropriate early discharge from intensive care unit: logistic regression triage model. *BMJ* 2001;322:1274–6.

7 Goldfrad C, Rowan K. Consequences of discharges from intensive care at night. *Lancet* 2000;355:1138–42.

8 Litton E, Ho KM, Lee KY et al. C-reactive protein concentration as a predictor of in-hospital mortality after ICU discharge: a nested case-control study. Intensive Care Med 2008;34:481–7.

9 NCEPOD. Knowing the Risk. A Review of the Peri-operative Care of Surgical Patients. London: National Confidential Enquiry into Patient Oucome and Death 2011.

10 NCEPOD. An acute problem. Nursing the elderly : in Hospital, Homes and the Community. London: National Confidential Enquiry into Patient Oucome and Death 2005..

11 NEPOD. Time to Intervene ? A Review of Patients who Underwent Cardiopulmonary Resuscitation as a Result of an In-hospital Cardiac Arrest. London: National Confidential Enquiry into Patient Oucome and Death 2012.

12 The Royal College of Surgeons. Emergency Surgery: Standards for unscheduled surgical care. London; 2011. Available from: https://www.rcseng.ac.uk/publications/docs/emergency-surgery-standards-for-unscheduled-care

13 Francis R. Report of the Mid Staffordshire NHS Foundation Trust Public Inquiry. London: The Stationary Office 2013. Available from: http://www.midstaffspublicinquiry.com/sites/default/files/report/Executive summary.pdf

14 Keogh B. Review into the quality of care and treatment provided by 14 hospital trusts in England: overview report. London: Hm Govt. 2013. Available from: http://www.nhs.uk/NHSEngland/bruce-keogh-review/Documents/outcomes/keogh-review-final-report.pdf

15 NCEPOD. Tracheostomy Care: On the Right Trach? London: National Confidential Enquiry into Patient Oucome and Death 2014.

16 Department of Health. Comprehensive Critical Care: A Review Of Adult Critical Care Services. London: Department of Health 2000.

17 Rowan K, Adam S, Ball C, et al. NIHR Service Delivery and Organisation programme. Evaluation of

For peer review only - http://bmjopen.bmj.com/site/about/guidelines.xhtml

outreach services in critical care. Southampton; NIHR 2004.

18 Gao H, Harrison DA, Parry GJ, et al. The impact of the introduction of critical care outreach services in England: a multicentre interrupted time-series analysis. *Crit Care* 2007 11:R113.

19 Forsberg A, Lindgren E, Engström Å. Being transferred from an intensive care unit to a ward: Searching for the known in the unknown. *Int J Nurs Pract* 2011;17:110–6.

20 Leith BA. Patients' and family members' perceptions of transfer from intensive care. *Hear Lung J Acute Crit Care* 1999;28:210–8.

21 Ludin SM, Arbon P, Parker S. Patients' transition in the intensive care units: concept analysis. *Intensive Crit Care Nurs* 2013;29:187–92.

22 Strahan EHE, Brown RJ. A qualitative study of the experiences of patients following transfer from intensive care. *Intensive Crit Care Nurs* 2005;21:160–71.

23 Mckinney A, Deeny P. Leaving the intensive care unit: A phenomenological study of the patients' experience. *Intensive Crit Care Nurs* 2002;18:320–31.

24 Odell M. The patient's thoughts and feelings about their transfer from intensive care to the general ward. *J Adv Nurs* 2000 31:322–9.

25 Field K, Prinjha S, Rowan K. "One patient amongst many": a qualitative analysis of intensive care unit patients' experiences of transferring to the general ward. *Crit Care* 2008;12:R21.

26 Lin F, Chaboyer W, Wallis M, et al. Factors contributing to the process of intensive care patient discharge: an ethnographic study informed by activity theory. *Int J Nurs Stud* 2013;50:1054–66.

27 Häggström M, Asplund K, Kristiansen L. Struggle with a gap between intensive care units and general wards. *Int J Qual Stud Health Well-being* 2009;4:181–92.

28 Häggström M, Asplund K, Kristiansen L. How can nurses facilitate patient's transitions from intensive care?. A grounded theory of nursing. *Intensive Crit Care Nurs* 2012;28:224–33.

29 Whittaker J, Ball C. Discharge from intensive care: a view from the ward. *Intensive Crit Care Nurs* 2000;16:135–43.

30 Martinez GH, Fernandez R, Casado MS, et al. Tracheostomy tube in place at intensive care unit discharge is associated with increased ward mortality. *Respir Care* 2009;54:1644–52.

31 Fernandez R, Bacelar N, Hernandez G, et al. Ward mortality in patients discharged from the ICU with tracheostomy may depend on patient's vulnerability. *Intensive Care Med* 2008;34:1878–82.

32 Araújo I, Gonçalves-Pereira J, Teixeira S, et al. Assessment of risk factors for in-hospital mortality after intensive care unit discharge. *Biomarkers* 2012;17:180–5.

33 Ho KM, Lee KY, Dobb GJ, et al. C-reactive protein concentration as a predictor of in-hospital mortality after ICU discharge: A prospective cohort study. *Intensive Care Med* 2008;34:481–7.

34 Ranzani OT, Prada LF, Zampieri FG, et al. Failure to reduce C-reactive protein levels more than 25% in the last 24 hours before intensive care unit discharge predicts higher in-hospital mortality: A cohort study. *J Crit Care* 2012;27:525.e9-15.

35 Gantner D, Bailey M, Huckson S, et al. Mortality related to after-hours discharge from intensive care in Australia and New Zealand. *Intensive Care Med* 2014;40:1528–35.

36 Beck DH, McQuillan P, Smith GB. Waiting for the break of dawn? The effects of discharge time, discharge TISS scores and discharge facility on hospital mortality after intensive care. *Intensive Care*

For peer review only

Med 2002;28:1287–93.

37 Pilcher D V, Duke GJ, George C, et al. After-hours discharge from intensive care increases the risk of readmission and death. *Anaesth Intensive Care* 2007;35:477–85.

38 Laupland KB, Misset B, Souweine B, et al. Mortality associated with timing of admission to and discharge from ICU: a retrospective cohort study. *BMC Health Serv Res* 2011;11:321.

39 Priestap F, Martin CM. Impact of intensive care unit discharge time on patient outcome. *Crit Care Med* 2006;34:2946–51.

40 Uusaro A, Kari A, Ruokonen E. The effects of ICU admission and discharge times on mortality in Finland. *Intensive Care Med* 2003;29:2144–8.

41 Pilcher D, Duke GJ, George C, et al. Pilcher After-hours discharge from ICU. *Anaesth Intensive Care* 2007;35:477–85.

42 Vollam S, Dutton S, Lamb S, et al. Out-of-hours discharge from intensive care, in-hospital mortality and intensive care readmission rates: a systematic review and meta-analysis. *Intensive Care Medicine* 2018;44:1115-1129.

43 Lawrence A, Havill JH. An audit of deaths occurring in hospital after discharge from the intensive care unit. *Anaesth Intensive Care* 1999;27:185–9.

44 McLaughlin N, Leslie GD, Williams TA, et al. Examining the occurrence of adverse events within 72 hours of discharge from the intensive care unit. *Anaesth Intensive Care* 2007;35:486–93.

45 Mayr VD, Dünser MW, Greil V, et al. Causes of death and determinants of outcome in critically ill patients. *Crit Care* 2006;10:R154.

46 Trivedi M, Ridley SA. Intermediate outcome of medical patients after intensive care. *Anaesthesia* 2001;56(9):841–6.

47 Denehy L, Skinner EH, Edbrooke L, et al. Exercise rehabilitation for patients with critical illness: a randomized controlled trial with 12 months of follow-up. *Crit Care* 2013;17:R156.

48 Jackson JC, Ely EW, Morey MC, et al. Cognitive and physical rehabilitation of intensive care unit survivors: results of the RETURN randomized controlled pilot investigation. *Crit Care Med* 2012;40:1088–97.

49 Elliott D, McKinley S, Alison J, et al. Health-related quality of life and physical recovery after a critical illness: a multi-centre randomised controlled trial of a home-based physical rehabilitation program. *Crit Care* 2011;15:R142.

50 Adler J, Malone D. Early mobilization in the intensive care unit: a systematic review. Cardiopulm Phys Ther J 2012;23:5–13.

51 Walsh TS, Salisbury LG, Merriweather JL, et al. Increased hospital-based physical rehabilitation and information provision after intensive care unit discharge: The RECOVER randomized clinical trial. *JAMA Internal Medicine,* 2015;175,:901-910.

52 NHS National Quality Board. National Quality Board Patient Experience Framework. 2012; Available from: http://www.institute.nhs.uk/patient_experience/guide/the_policy_framework.html

53 Vincent C, Neale G, Woloshynowych M. Adverse events in British hospitals: preliminary retrospective record review. *BMJ* 2001;322:517–9.

54 NCEPOD. *Caring to the End? A Review of the Care of Patients Who Died Within Four Days of*

For peer review only - http://bmjopen.bmj.com/site/about/guidelines.xhtml

Hospital Admission. London: National Confidential Enquiry into Patient Oucome and Death 2010.

55 Royal College of Physicians. National Mortality Case Record Review Programme: structured case note review data collection. London: Royal College of Physicians 2017.

56 Hogan H, Healey F, Neale G, et al. Preventable deaths due to problems in care in English acute hospitals: a retrospective case record review study. *BMJ Qual Saf* 2012;21:737–45.

57 Hutchinson A, Coster JE, Cooper KL, et al. A structured judgement method to enhance mortality case note review: development and evaluation. *BMJ Qual Saf*. 2013;22:1032–40.

58 Hogan H, Healey F, Neale G, et al. Learning from preventable deaths: exploring case record reviewers' narratives using change analysis. *J R Soc Med* 2014;107:365–75.

59 Odell M, Gerber K, Gager M. Activated Critical Care Outreach. *Methods* 2010;19:599-602.

60 Rance S, McCourt C, Rayment J, et al. Women's safety alerts in maternity care: is speaking up enough? *BMJ Qual Saf* 2013;22:348–55.

61 Ward JK, Armitage G. Can patients report patient safety incidents in a hospital setting? A systematic review. *BMJ Qual Saf* 2012;21:685–99.

62 Bryant A, Charmaz K. The SAGE Handbook of Grounded Theory. London: Sage Publications 2007.

63 Creswell J. Qualitative Inquiry And Research Design: Choosing Among Five Approaches. London: Sage Publications 2012.

64 Pope C, Ziebland S, Mays N. Qualitative research in health care. Analysing qualitative data. *BMJ* 2000;320:114–6.

65 Coyne IT. Sampling in qualitative research. Purposeful and theoretical sampling; merging or clear boundaries? *J Adv Nurs* 1997;26:623–30.

66 Parkes CM. Guidelines for conducting ethical bereavement research. *Death Stud* 1995;19:171–81.

67 Bentley B, O'Connor M. Conducting research interviews with bereaved family carers: when do we ask? *J Palliat Med* 2014;18:241–5.

68 Chapple A, Ziebland S, Hawton K. Taboo and the different death? Perceptions of those bereaved by suicide or other traumatic death. *Sociol Heal Illn* 2015;37:610–25.

69 Walker D, Myrick F. Grounded theory: an exploration of process and procedure. *Qual Health Res* 2006;16:547–59.

70 Hinton L, Locock L, Knight M. Maternal critical care: what can we learn from patient experience? A qualitative study. *BMJ Open* 2015;5:e006676.

71 Ziebland S, McPherson A. Making sense of qualitative data analysis: an introduction with illustrations from DIPEx (personal experiences of health and illness). Med Educ. 2006;40(5):405–14.

72 Medical Research Council. A framework for development and evaluation of RCTs for complex interventions to improve health. London; MRC 2000.

73 Chan A-W, Tetzlaff JM, Altman DG, et al. SPIRIT 2013 Statement: Defining standard protocol items for clinical trials. Ann Intern Med 2013;158:200-207.

For peer review only

FIGURE LEGENDS:

Figure 1. Primary data collection

Figure 2. Modelling the intervention

For peer review only

For peer review only - http://bmjopen.bmj.com/site/about/guidelines.xhtml

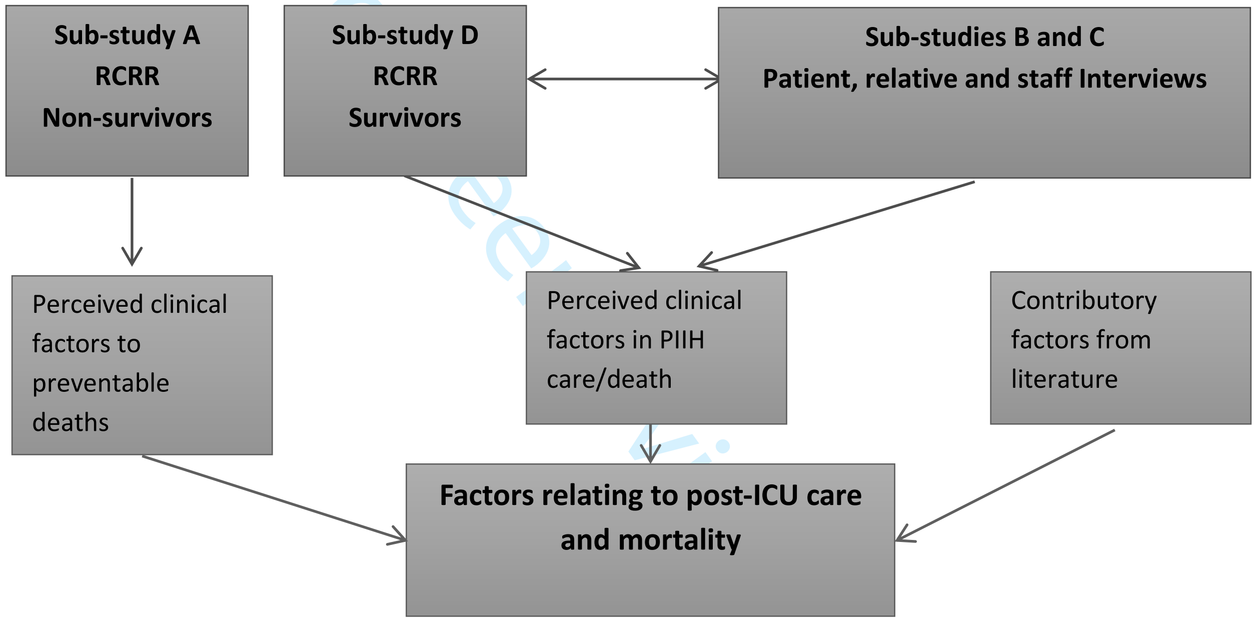

Figure 1. Work flow

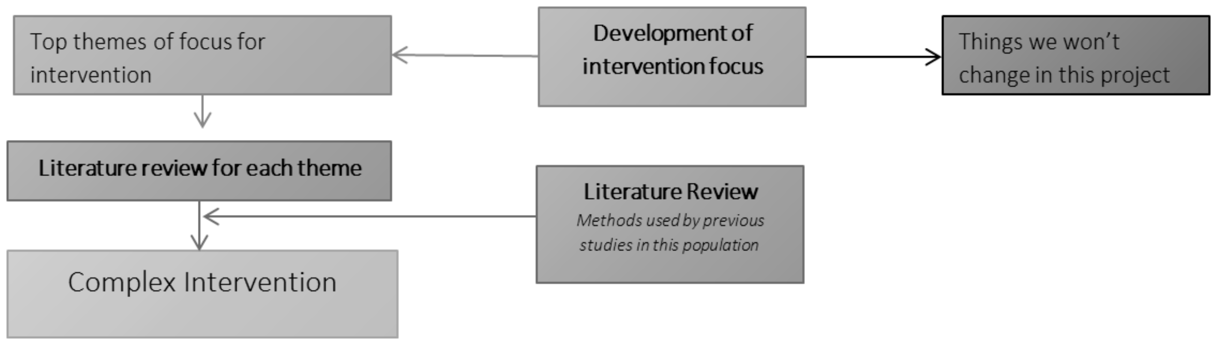

For peer review only

SPIRIT

STANDARD PROTOCOL ITEMS: RECOMMENDATIONS FOR INTERVENTIONAL TRIALS

SPIRIT 2013 Checklist: Recommended items to address in a clinical trial protocol and related documents*

Section/item	ItemNo	Description	
**Administrative information**			
Title	1	Descriptive title identifying the study design, population, interventions, and, if applicable, trial acronym	p1
Trial registration	2a	Trial identifier and registry name. If not yet registered, name of intended registry	Abstract and p 9
	2b	All items from the World Health Organization Trial Registration Data Set	As per registry
Protocol version	3	Date and version identifier	p9
Funding	4	Sources and types of financial, material, and other support	p10
Roles and responsibilities	5a	Names, affiliations, and roles of protocol contributors	p1
	5b	Name and contact information for the trial sponsor	p9
	5c	Role of study sponsor and funders, if any, in study design; collection, management, analysis, and interpretation of data; writing of the report; and the decision to submit the report for publication, including whether they will have ultimate authority over any of these activities	n/a
	5d	Composition, roles, and responsibilities of the coordinating centre, steering committee, endpoint adjudication committee, data management team, and other individuals or groups overseeing the trial, if applicable (see Item 21a for data monitoring committee)	p3
**Introduction**			
Background and rationale	6a	Description of research question and justification for undertaking the trial, including summary of relevant studies (published and unpublished) examining benefits and harms for each intervention	p3
	6b	Explanation for choice of comparators	n/a
Objectives	7	Specific objectives or hypotheses	p4
Trial design	8	Description of trial design including type of trial (eg, parallel group, crossover, factorial, single group), allocation ratio, and framework (eg, superiority, equivalence, noninferiority, exploratory)	p4

For peer review only

Methods: Participants, interventions, and outcomes

Study setting	9	Description of study settings (eg, community clinic, academic hospital) and list of countries where data will be collected. Reference to where list of study sites can be obtained	p5
Eligibility criteria	10	Inclusion and exclusion criteria for participants. If applicable, eligibility criteria for study centres and individuals who will perform the interventions (eg, surgeons, psychotherapists)	P4-5
Interventions	11a	Interventions for each group with sufficient detail to allow replication, including how and when they will be administered	For interviews: p5
	11b	Criteria for discontinuing or modifying allocated interventions for a given trial participant (eg, drug dose change in response to harms, participant request, or improving/worsening disease)	n/a
	11c	Strategies to improve adherence to intervention protocols, and any procedures for monitoring adherence (eg, drug tablet return, laboratory tests)	n/a
	11d	Relevant concomitant care and interventions that are permitted or prohibited during the trial	n/a
Outcomes	12	Primary, secondary, and other outcomes, including the specific measurement variable (eg, systolic blood pressure), analysis metric (eg, change from baseline, final value, time to event), method of aggregation (eg, median, proportion), and time point for each outcome. Explanation of the clinical relevance of chosen efficacy and harm outcomes is strongly recommended	p8
Participant timeline	13	Time schedule of enrolment, interventions (including any run-ins and washouts), assessments, and visits for participants. A schematic diagram is highly recommended (see Figure)	n/a
Sample size	14	Estimated number of participants needed to achieve study objectives and how it was determined, including clinical and statistical assumptions supporting any sample size calculations	p7
Recruitment	15	Strategies for achieving adequate participant enrolment to reach target sample size	p6

Methods: Assignment of interventions (for controlled trials)

Allocation:

For peer review only - http://bmjopen.bmj.com/site/about/guidelines.xhtml

Sequence generation	16a	Method of generating the allocation sequence (eg, computer-generated random numbers), and list of any factors for stratification. To reduce predictability of a random sequence, details of any planned restriction (eg, blocking) should be provided in a separate document that is unavailable to those who enrol participants or assign interventions	n/a
Allocation concealment mechanism	16b	Mechanism of implementing the allocation sequence (eg, central telephone; sequentially numbered, opaque, sealed envelopes), describing any steps to conceal the sequence until interventions are assigned	n/a
Implementation	16c	Who will generate the allocation sequence, who will enrol participants, and who will assign participants to interventions	n/a
Blinding (masking)	17a	Who will be blinded after assignment to interventions (eg, trial participants, care providers, outcome assessors, data analysts), and how	n/a
	17b	If blinded, circumstances under which unblinding is permissible, and procedure for revealing a participant's allocated intervention during the trial	n/a
**Methods: Data collection, management, and analysis**			
Data collection methods	18a	Plans for assessment and collection of outcome, baseline, and other trial data, including any related processes to promote data quality (eg, duplicate measurements, training of assessors) and a description of study instruments (eg, questionnaires, laboratory tests) along with their reliability and validity, if known. Reference to where data collectionforms can be found, if not in the protocol	p4-5
	18b	Plans to promote participant retention and complete follow-up, including list of any outcome data to be collected for participants who discontinue or deviate from intervention protocols	n/a
Data management	19	Plans for data entry, coding, security, and storage, including any related processes to promote data quality (eg, double data entry; range checks for data values). Reference to where details of data management procedures can be found, if not in the protocol	p7
Statistical methods	20a	Statistical methods for analysing primary and secondary outcomes. Reference to where other details of the statistical analysis plan can be found, if not in the protocol	p7-8
	20b	Methods for any additional analyses (eg, subgroup and adjusted analyses)	p7-8

For peer review only - http://bmjopen.bmj.com/site/about/guidelines.xhtml

	20c	Definition of analysis population relating to protocol non-adherence (eg, as randomised analysis), and any statistical methods to handle missing data (eg, multiple imputation)	n/a
**Methods: Monitoring**			
Data monitoring	21a	Composition of data monitoring committee (DMC); summary of its role and reporting structure; statement of whether it is independent from the sponsor and competing interests; and reference to where further details about its charter can be found, if not in the protocol. Alternatively, an explanation of why a DMC is not neede	n/a
	21b	Description of any interim analyses and stopping guidelines, including who will have access to these interim results and make the final decision to terminate the trial	n/a
Harms	22	Plans for collecting, assessing, reporting, and managing solicited and spontaneously reported adverse events and other unintended effects of trial interventions or trial conduct	p9
Auditing	23	Frequency and procedures for auditing trial conduct, if any, and whether the process will be independent from investigators and the sponsor	n/a
**Ethics and dissemination**			
Research ethics approval	24	Plans for seeking research ethics committee/institutional review board (REC/IRB) approval	p9
Protocol amendments	25	Plans for communicating important protocol modifications (eg, changes to eligibility criteria, outcomes, analyses) to relevant parties (eg, investigators, REC/IRBs, trial participants, trial registries, journals, regulators)	n/a
Consent or assent	26a	Who will obtain informed consent or assent from potential trial participants or authorised surrogates, and how (see Item 32)	p7
	26b	Additional consent provisions for collection and use of participant data and biological specimens in ancillary studies, if applicable	n/a
Confidentiality	27	How personal information about potential and enrolled participants will be collected, shared, and maintained in order to protect confidentiality before, during, and after the trial	p7
Declaration of interests	28	Financial and other competing interests for principal investigators for the overall trial and each study site	p10
Access to data	29	Statement of who will have access to the final trial dataset, and disclosure of contractual agreements that limit such access for investigators	n/a

For peer review only

Ancillary and post-trial care	30	Provisions, if any, for ancillary and post-trial care, and for compensation to those who suffer harm from trial participation	p9-10
Dissemination policy	31a	Plans for investigators and sponsor to communicate trial results to participants, healthcare professionals, the public, and other relevant groups (eg, via publication, reporting in results databases, or other data sharing arrangements), including any publication restrictions	p10
	31b	Authorship eligibility guidelines and any intended use of professional writers	p10
	31c	Plans, if any, for granting public access to the full protocol, participant-level dataset, and statistical code	n/a
**Appendices**			
Informed consent materials	32	Model consent form and other related documentation given to participants and authorised surrogates	p7 (on trial registry)
Biological specimens	33	Plans for collection, laboratory evaluation, and storage of biological specimens for genetic or molecular analysis in the current trial and for future use in ancillary studies, if applicable	n/a

*It is strongly recommended that this checklist be read in conjunction with the SPIRIT 2013 Explanation & Elaboration for important clarification on the items. Amendments to the protocol should be tracked and dated. The SPIRIT checklist is copyrighted by the SPIRIT Group under the Creative Commons "Attribution-NonCommercial-NoDerivs 3.0 Unported" license.

For peer review only
