# Supplementary material for: Protocol for a mixed-methods exploratory investigation of care following intensive care discharge: the REFLECT study
Source: BMJ Open. 2019 Jan 25;9(1):e027838. doi: 10.1136/bmjopen-2018-027838 (PMC6347880; doi:10.1136/bmjopen-2018-027838)
Supplement: Reviewer comments [file bmjopen-2018-027838.reviewer_comments.pdf]

PEER REVIEW HISTORY

BMJ Open publishes all reviews undertaken for accepted manuscripts. Reviewers are asked to complete a checklist review form (**http://bmjopen.bmj.com/site/about/resources/checklist.pdf**) and are provided with free text boxes to elaborate on their assessment. These free text comments are reproduced below.

ARTICLE DETAILS

**TITLE (PROVISIONAL)**	Protocol for a mixed methods exploratory investigation of care following intensive care discharge: the REFLECT study.
**AUTHORS**	Vollam, Sarah; Gustafson, Owen; Hinton, Lisa; Morgan, Lauren; Pattison, Natalie; Thomas, Hilary; Young, Duncan; Watkinson, Peter

This article was not externally reviewed at BMJ Open. Protocols that have been independently assessed prior to submission to BMJ Open are usually fast-tracked to publication on the grounds that further substantial changes will not be possible. This independent assessment will usually be external, independent review for both a substantial grant award from a non-commercial or government funder and ethics approval.
